# Cascade Valorisation of Lemon-Processing Residues (Part I): Current Trends in Green Extraction Technologies and High-Value Bioactive Recovery

**DOI:** 10.3390/foods15030491

**Published:** 2026-02-01

**Authors:** Jimmy Núñez-Pérez, Jhomaira L. Burbano-García, Rosario Espín-Valladares, Marco V. Lara-Fiallos, Juan Carlos DelaVega-Quintero, Marcelo Cevallos-Vallejos, José-Manuel Pais-Chanfrau

**Affiliations:** 1 School of Agro-Industrial Engineering, Faculty of Engineering in Agricultural and Environmental Sciences (FICAYA), Universidad Técnica del Norte, Ibarra 100105, Ecuador; jlburbano@utn.edu.ec (J.L.B.-G.); rcespin@utn.edu.ec (R.E.-V.); mvlara@utn.edu.ec (M.V.L.-F.); jcdelavega@utn.edu.ec (J.C.D.-Q.); 2 Faculty of Engineering in Agricultural and Environmental Sciences (FICAYA), Universidad Técnica del Norte, Ibarra 100105, Ecuador; mcevallos@utn.edu.ec

**Keywords:** *Citrus × limon*, lemon-peel waste, biorefinery, cascade valorisation, ultrasound-assisted extraction, microwave-assisted extraction, supercritical fluid extraction, pectin, D-limonene, nanocrystalline cellulose

## Abstract

The global citrus-processing industry generates 15–32 million tonnes of waste annually. Lemon-processing residues—peels, seeds, and pomace—constitute 45–55% of fruit mass and harbour high-value bioactive compounds amenable to cascade valorisation. This review (Part I of a two-part series) examines green extraction technologies for recovering bioactive compounds from lemon waste streams. Following bibliometric analysis of 847 publications (2003–2025), this work delineates the compositional heterogeneity of lemon fractions and establishes a hierarchical framework for value-added products encompassing essential oils, pectin, polyphenols, seed oils, citric acid, industrial enzymes, α-cellulose, and nanocrystalline cellulose. Four sustainable extraction methodologies are systematically evaluated: ultrasound-assisted extraction, microwave-assisted extraction, supercritical CO_2_ extraction, and enzyme-assisted extraction. Comparative assessment demonstrates yield improvements of 16–112% over conventional approaches, processing-time reductions of 89–98%, and energy savings up to 95%. Critical research gaps include fragmented single-product valorisation, insufficient techno-economic assessment, and limited industrial-scale validation. Integrated cascade biorefineries employing sequential green extraction protocols offer economically viable pathways for transforming lemon waste into diversified revenue streams. Industrial implementation, circular-economy integration, and techno-economic feasibility are addressed in Part II.

## 1. Introduction

### 1.1. Global Food, Agricultural, and Agro-Industrial Waste: Volumes and Economic Implications

The global food system generates staggering quantities of agricultural, food, and agro-industrial waste, representing one of the most pressing sustainability challenges of contemporary society. According to recent estimates by the Food and Agriculture Organization (FAO), approximately 13.2% of food produced globally, amounting to over 1.05 billion tonnes annually, is lost in the supply chain after harvest and before reaching retail markets, with an additional 19% wasted at the retail, food service, and household levels [1]. The aggregate volume of food loss and waste thus exceeds 1.3 billion tonnes per year [2], a figure projected to increase to 2.2 billion tonnes in 2025 if current trends persist [3,4]. Agricultural residues constitute an even larger waste stream, with crop residues alone contributing millions of tonnes annually to the global biomass waste pool. These enormous quantities of discarded organic matter impose substantial environmental burdens through greenhouse gas emissions—accounting for 8–10% of global emissions—alongside considerable losses of freshwater resources, energy, and productive agricultural land [1].

The economic ramifications of this widespread wastage are equally profound. Globally, food loss and waste incur direct economic costs estimated at approximately USD 940 billion annually [5], with regional disparities evident: high-income countries account for approximately USD 680 billion in losses, whilst low- and middle-income countries bear costs approaching USD 310 billion [2]. In the United States alone, 40% of food production is lost or wasted annually, representing an economic loss of USD 218 billion—equivalent to approximately 1.3% of the nation’s gross domestic product [6]. At the producer level, food waste throughout the supply chain results in financial losses of 15–30% of total production value for farmers, processors, and retailers. The hospitality and food service sectors experience particularly acute impacts, with profit losses of up to 4% directly attributable to wasted food [6]. These losses extend beyond mere monetary valuations; they represent squandered investments in water, energy, labour, and land resources that could otherwise contribute to food security and economic development.

Agro-industrial waste streams present both a significant challenge and an opportunity within the circular-economy framework. The processing of agricultural commodities generates vast quantities of by-products—including peels, seeds, pomace, bagasse, husks, and pulp—that traditionally receive inadequate valorisation [7]. Optimised valorisation strategies, such as integrated biorefinery approaches that process potato-peel waste into multiple bioproducts, have demonstrated potential revenues exceeding USD 6300 per tonne of dry waste—substantially surpassing conventional disposal or single-product recovery pathways [8].

Addressing these challenges requires comprehensive circular-bioeconomy strategies that transform waste liabilities into economic assets [9]. International commitments, including the United Nations Sustainable Development Goal 12.3, target a 50% reduction in per capita food waste by 2030, alongside minimising losses throughout production and supply chains [10]. Achieving these ambitious targets necessitates coordinated action across multiple scales: from farm-level adoption of improved harvesting and post-harvest handling practices to industrial implementation of advanced biorefinery technologies and consumer-level behavioural modifications [11]. Investment analyses suggest that directing USD 14 billion annually towards food-waste reduction solutions over the next decade could yield substantial economic returns—estimated at USD 14 for every dollar invested—while simultaneously reducing greenhouse gas emissions by 75 million metric tonnes and redirecting food equivalent to four billion meals towards populations experiencing food insecurity [6]. Within this broader context, the development of cascading-valorisation strategies for specific agricultural and agro-industrial waste streams, such as lemon-processing residues, is a critical component of the transition towards more sustainable, circular food-production systems [3,4].

### 1.2. Global Lemon Production and Waste Generation

**Figure 1:**
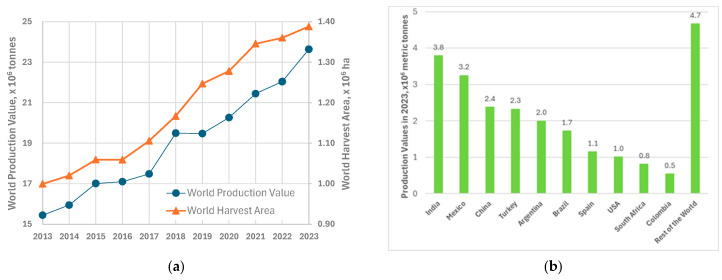
Global lemon and lime production trends (2013–2023). (**a**) Evolution of total production volume (million metric tonnes) and cultivated area (million hectares); (**b**) Geographical distribution of lemon production by major producing countries (annual production in million tonnes).

Global lemon (*Citrus × limon* or *Citrus limon*) and lime (*Citrus* × *aurantiifolia*, *Citrus* × *latifolia*, *Citrus hystrix*, and *Citrus glauca*) production reached approximately 23.64 million metric tonnes in 2023 [1], representing a notable increase from 22.04 million tonnes in the previous year. This upward trajectory continues a long-term growth pattern, with production expanding nearly 53% from 15.4 million tonnes in 2013 to 23.64 million tonnes by 2023 (Figure 1a). The cultivated area for lemon and lime production has similarly expanded, growing from 999,642 hectares in 2013 to over 1.39 million hectares in 2023 (Figure 1a).

The geographical distribution of lemon production exhibits significant regional concentration. India remains the world’s largest producer, with 3.8 million tonnes annually, followed by Mexico, China, Turkey, Argentina, Brazil, Spain, the USA, South Africa, and Colombia (Figure 1b).

Recent forecasts for the 2024/25 global lemon and lime production amounted to around 21.5 million metric tonnes, a decrease from 2023. Also, the 2025 season indicates dynamic shifts in regional production patterns. Mexican output is projected to reach 3.5 million tonnes, an 8% increase attributed to favourable weather conditions during the bloom and fruit-set phases. Conversely, significant production declines are anticipated in several major producing regions: Turkish production is forecast to decrease by over 30% to 1.6 million tonnes due to heat stress during flowering, whilst European Union production is expected to decline by 14% to approximately 1.5 million tonnes. Spanish lemon production specifically decreased by 14.7% to 866,654 tonnes, primarily due to adverse climatic events. Argentine production fell by 70,000 tonnes to 1.4 million tonnes owing to insufficient rainfall during critical growth periods. In contrast, South African production has grown, with forecasts indicating a 7% increase to 780,000 tonnes [12].

**Figure 2:**
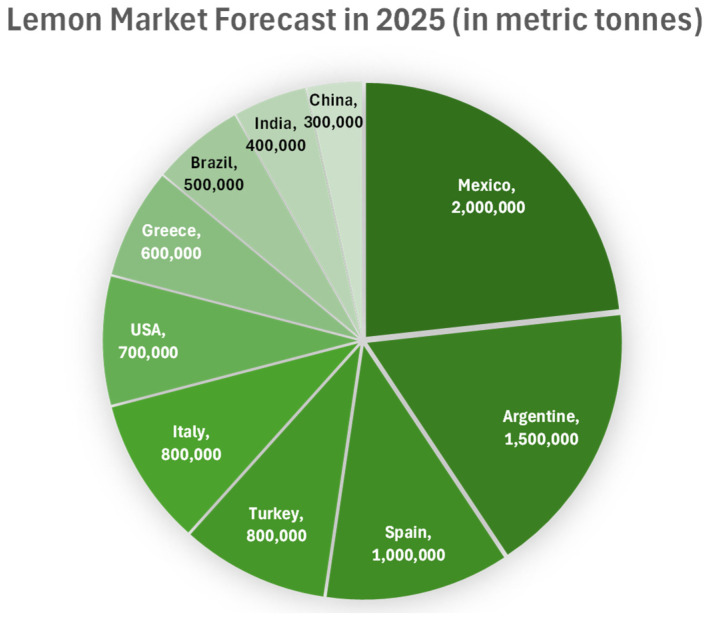
Market dynamics and growth projections for the global lemon industry. Overview of key drivers, market valuation forecasts, and regional growth trends shaping the lemon sector through 2025.

The international lemon sector is experiencing steady growth, driven by increased demand for lemon-derived products worldwide. Estimates from Market Research Future indicate that the global lemon market is projected to reach $8.5 billion by 2025, with a compound annual growth rate of 4.5% over the forecast period [13]. This upward trend is attributable to the increasing popularity of lemon-containing beverages, the recognised health benefits of lemon consumption, and the broader integration of lemons into culinary practices (Figure 2).

The global lemon sector is forecast to maintain a robust growth trajectory over the coming years, primarily driven by rising consumer preferences for natural, health-oriented products [13]. The surging popularity of lemon-containing beverages [14], the well-documented health benefits of lemon intake [15], and the increasingly diverse culinary applications [16] are expected to drive market growth. Industry analysts predict a notable increase in both lemon production and consumption across the Asia-Pacific region, with China and India at the forefront of this growth [13]. Furthermore, advances in lemon cultivation and processing technologies are expected to drive industry growth, offering stakeholders novel opportunities to innovate and diversify their product portfolios. By strategically aligning with these prevailing trends, businesses in the lemon industry can benefit from increased demand and strengthen their competitive position in the global market [13].

The citrus-processing industry, encompassing lemon valorisation, generates substantial volumes of organic waste. Globally, the citrus-processing sector produces over 15 million tonnes of waste by-products annually, primarily peels, pulp, and seeds. The Food and Agriculture Organization estimates that approximately 10 million metric tonnes of citrus-fruit-processing waste—including lemon-derived materials—are generated each year worldwide. This waste represents a considerable environmental challenge when inadequately managed, whilst simultaneously offering significant opportunities for valorisation within circular economic frameworks.

**Figure 3:**
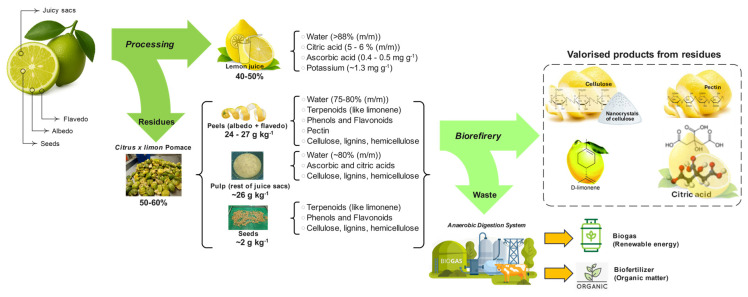
Compositional distribution of lemon-processing waste fractions. Representation of the relative proportions (g·kg^−1^) of flavedo, albedo, pulp, and seeds generated during industrial-scale lemon-juice-extraction operations, alongside moisture content and key biochemical components.

Citrus-processing waste typically comprises 50–60% of the original fresh-fruit mass. More specifically, lemon juice production generates waste streams wherein peels account for 50–55% of total fruit mass, seeds represent 20–40%, whilst pulp and other residues constitute the remainder [17]. Alternative estimates indicate that lemon-processing by-products may represent up to 26 g·kg^−1^ pulp, 17 g·kg^−1^ albedo, 10 g·kg^−1^ flavedo, and 2 g·kg^−1^ seeds in juice-extraction operations (Figure 3).

The compositional profile of lemon waste fractions reveals substantial heterogeneity. Lemon peel exhibits a moisture content of 75.30 ± 10.20%, with high crude fibre content (57.0 ± 10.0%) and crude protein (10.2 ± 3.7%). The peel also contains 2.22 ± 0.61% crude fat and 3.33 ± 0.50% total ash. Dried lemon peel demonstrates distinct compositional characteristics, containing approximately 13% pectin (dry-weight basis), 7.56% lignin, 23.06% cellulose, and 8.09% hemicellulose. In contrast, lemon pulp contains elevated moisture levels (85.7 ± 0.0%) but substantially lower concentrations of crude fibre (4.9 ± 0.0%) and protein (8.6 ± 0.0%). Lemon seeds are characterised by exceptionally high oil fat content (52.0%), with comparatively lower fibre (5.5%), protein (3.1%), and ash (2.5%) levels [18,19,20].

Fresh citrus by-products, including lemon residues, are particularly rich in fermentable carbohydrates (approximately 28.5%) and have a low lignin content (approximately 3.5%), making them suitable substrates for various biotransformation processes [18,20]. The albedo (inner white-peel layer) serves as the primary source of pectin [21], whilst the flavedo (outer coloured layer) contains substantial concentrations of terpenoids, particularly D-limonene, which constitutes the predominant component of lemon essential oils [22,23].

### 1.3. Environmental Challenges and the Circular-Economy Concept

The substantial waste generated by lemon processing presents both environmental challenges and economic opportunities [24]. Traditional disposal methods—including landfilling and incineration—prove inadequate, generating harmful methane emissions, producing malodorous compounds, consuming a considerable amount of energy, and exhibiting slow degradation kinetics [25,26]. Furthermore, unauthorised disposal of citrus-processing waste can contaminate soil and aquatic ecosystems, particularly in regions with insufficient dilution capacity [26].

Contemporary approaches emphasise waste valorisation strategies that transform lemon by-products into renewable chemicals, fuels, and energy carriers within integrated biorefinery frameworks [26,27,28].

The biorefinery concept applied to lemon waste encompasses the sequential or simultaneous extraction of multiple value-added fractions, including, among others, essential oils (rich in D-limonene) [22,29], pectin (a valuable hydrocolloid) [30,31], phenolic compounds (with antioxidant properties) [32], dietary fibres [33], cellulose [34], and cellulose nanocrystals [34,35]. Advanced extraction technologies—including microwave-assisted extraction, ultrasound-assisted extraction, supercritical fluid extraction, and pulsed electric field treatment—enable the efficient recovery of these bioactive compounds while minimising energy consumption and solvent usage [36,37,38].

The integration of circular-economy principles into lemon-processing operations facilitates the transition from linear “take–make–dispose” models to closed-loop systems in which waste streams become feedstocks for subsequent production processes [18,39]. Sequential extraction protocols exemplify this approach: after essential-oil recovery, the remaining solid residues can be utilised for pectin extraction [37,40,41,42], with the subsequent residual biomass serving as a raw material for cellulose and cellulose nanocrystal production [41,43]. Such cascading-valorisation strategies maximise resource-utilisation efficiency, reduce environmental impact, and generate multiple revenue streams, thereby enhancing the economic viability and sustainability of lemon-based biorefineries [21].

The development of lemon biorefineries aligns with Sustainable Development Goal 12, which targets a 50% reduction in global per capita food waste by 2030 and aims to minimise losses throughout supply chains [27,44,45]. By transforming lemon-processing waste into high-value products—including nutraceuticals, pharmaceutical intermediates, cosmetic ingredients, food additives, biomaterials, and renewable-energy carriers—these biorefinery systems contribute to environmental sustainability, food security, and regional economic development in lemon-producing areas [18,20].

### 1.4. Bibliometric Analysis of Research Landscape (2003–2025)

To systematically assess the current state of knowledge and identify research trends in lemon-waste valorisation, a comprehensive bibliometric analysis was conducted using the Web of Science (WoS) Core Collection database. The search strategy employed the query: (“Lemon” OR “*Citrus × limon*”) AND “Waste Valorisation” (including variant spellings: valorization, valorization, utilisation), covering publications from 2003 to October 2025. This 22-year period captures the evolution of research interest in citrus biorefinery concepts, from early waste-management approaches to contemporary circular-economy frameworks [46,47].

The retrieved dataset comprised 847 publications, encompassing research articles, reviews, conference proceedings, and book chapters. To visualise the intellectual structure and thematic clusters within this research domain, a bibliometric network analysis was performed using VOSviewer (version 1.6.19) [48], focusing on the co-occurrence of author keywords with a minimum threshold of five occurrences. VOSviewer has been extensively utilised in bibliometric studies across diverse fields due to its ability to generate distance-based visualisations where spatial proximity reflects conceptual relatedness [49]. This approach reveals the conceptual landscape of research on the valorisation of lemon waste and identifies both mature research areas and emerging frontiers.

#### 1.4.1. Network Structure and Thematic Clusters

**Figure 4:**
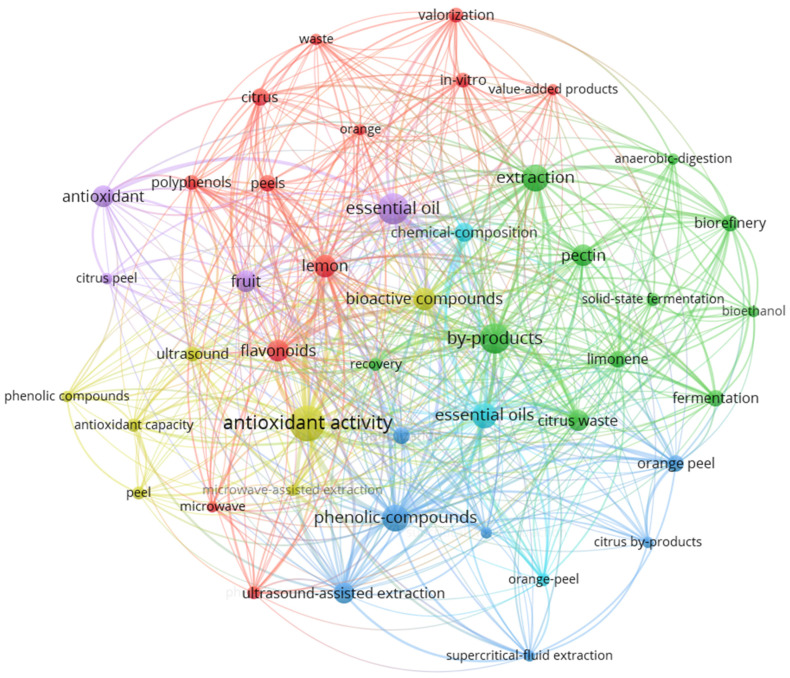
Bibliometric network visualisation of research on lemon-waste valorisation (2003–2025). Co-occurrence network of author keywords (minimum threshold: 5 occurrences) generated using VOSviewer, revealing thematic clusters, research priorities, and interconnections within the scientific literature on citrus-waste biorefinery concepts. Node size reflects keyword frequency; proximity indicates conceptual relatedness.

The co-occurrence network analysis (Figure 4) reveals a complex, interconnected research landscape organised around several distinct yet overlapping thematic clusters, each represented by colour-coded groupings in the visualisation. This network provides insights into the dominant research paradigms and evolving priorities within citrus-waste valorisation.

#### 1.4.2. Core Research Themes

**Bioactive Compounds and Antioxidant Activity (Purple-Blue Cluster):** The most densely interconnected cluster centres on the extraction and characterisation of bioactive compounds from lemon residues. Key nodes include “Bioactive Compounds & Antioxidants”, “Extraction Technologies”, “Citrus Waste & By-Products”, “Biorefinery & Value Addition”, and “Fermentation & Biotransformation” (the largest node, indicating high research frequency); also, “antioxidant activity”, “phenolic compounds”, “polyphenols”, “flavonoids”, and “antioxidant capacity”. This cluster reflects sustained research interest in the nutraceutical and functional-food applications of lemon by-products [16,50,51], with strong connections to extraction methodologies. The prominence of “antioxidant activity” as a central node demonstrates that bioactivity assessment remains the primary validation metric for extraction research. Lemon-peel phenolic compounds, particularly flavonoids such as hesperidin and eriocitrin, have been extensively characterised for their antioxidant, antimicrobial, and potential health-promoting properties [16].

**Extraction Technologies (Yellow-Orange Cluster):** A substantial research focus on green and efficient extraction methodologies is evident, with prominent nodes for “ultrasound-assisted extraction”, “microwave-assisted extraction”, and “supercritical fluid extraction”. The term “essential oil” appears as a central hub, connecting extraction technologies with product applications. This cluster demonstrates the field’s evolution beyond conventional solvent extraction toward environmentally sustainable and efficient processes. The strong connectivity between extraction methods and “essential oil” indicates that process innovation has been predominantly driven by the recovery of volatile compounds, particularly D-limonene, which comprises 90–95% of the composition of citrus essential oils [38,52,53,54].

**Citrus Waste and By-Products (Red-Pink Cluster):** This cluster encompasses the terminology for feedstock and residues, including “citrus waste”, “orange peel”, “citrus peel”, “citrus by-products”, and “lemon peel”. The inclusion of multiple citrus species (particularly orange) within the lemon-focused search reflects the transferability of valorisation strategies across citrus genera. The terms “peels”, “peel”, and “fruit” appear as significant nodes, emphasising that peel fractions are the primary focus of research on citrus-waste valorisation. Citrus peels account for 50–55% of total fruit weight and are rich in pectin, cellulose, essential oils, and bioactive compounds, making them ideal feedstocks for biorefinery applications [35,55,56]. Notably, “waste” appears as a central connecting node, bridging feedstock characterisation with valorisation approaches.

**Biorefinery and Value Addition (Green Cluster):** Research aligned with circular-economy principles is represented by nodes including “biorefinery”, “valorisation”, “value-added products”, “in vitro”, and “extraction”. The term “biorefinery” appears with moderate frequency but shows strong connections to multiple clusters, suggesting its role as an integrative concept rather than a standalone research area [20,57,58]. The presence of “pectin” and “limonene” as distinct nodes within this cluster indicates that these products are recognised as key value streams [22,35,59]. A recent industry report valued the global pectin market at about USD 1.17 billion in 2024 and projects an expansion to over USD 2.2 billion by 2033, with citrus-derived pectin accounting for the dominant share of production and use [60]. However, the relatively modest size of these nodes compared to “antioxidant activity” suggests that industrial product research remains less prominent than bioactivity studies.

**Fermentation and Biotransformation (Cyan-Teal Cluster):** A smaller but distinct cluster addresses biological valorisation approaches, including “anaerobic digestion”, “fermentation”, “bioethanol”, and “solid-state fermentation”. The term “fermentation” serves as a bridge between waste treatment and the production of value-added biochemicals [54,61,62]. This cluster’s moderate connectivity to the leading network suggests that while biotechnological approaches are established, they remain somewhat peripheral to the dominant extraction-focused research paradigm. Notably, D-limonene present in citrus residues can inhibit anaerobic digestion processes, necessitating its removal before biological treatment [63].

**Chemical Composition and Characterisation (Light Blue Cluster):** Analytical and compositional studies form another recognisable theme, with nodes including “chemical composition”, “recovery”, and connections to specific compound classes. This cluster reflects the fundamental characterisation work underpinning valorisation strategies. Comprehensive analyses have documented that citrus waste contains 80–90% moisture, with dry matter comprising soluble sugars (glucose, fructose, sucrose), structural polysaccharides (pectin, cellulose, hemicellulose), essential oils, phenolic compounds, and minerals [64,65]. However, this cluster’s relatively dispersed nature suggests that compositional analysis is typically integrated within broader valorisation studies rather than pursued as an isolated research objective.

#### 1.4.3. Emerging Research Frontiers

Several terms appear with increasing frequency in recent years, suggesting emerging research directions that merit attention for future biorefinery development:

• **Circular-Economy Integration:** The explicit appearance of terms related to circular-economy principles, though not yet forming a large node, indicates growing recognition of system-level thinking beyond single-product valorisation. Recent life-cycle assessment (LCA) studies have demonstrated that processing citrus residues in a biorefinery configuration offers superior environmental performance compared to conventional disposal practices, reducing global warming potential by 81–89% [66].
• **Nanocellulose and Advanced Materials:** While “nanocrystalline cellulose” does not appear as a significant node in the current network, related terms suggest nascent interest in advanced cellulosic materials from citrus residues, representing a high-value product frontier. Recent studies have successfully isolated cellulose nanocrystals (CNCs) from lemon seeds using sulphuric acid hydrolysis and oxidation methods, achieving yields of 17–19% and producing rod-like morphologies suitable for nanocomposite reinforcement applications [34,67].
• **Multi-Product Cascades:** The co-occurrence of multiple product terms (pectin, limonene, essential oils, citric acid) within interconnected clusters suggests growing awareness of cascade-valorisation concepts, though explicit cascade terminology remains limited in the current literature. Integrated approaches for extracting essential oils before pectin recovery have been demonstrated to improve both product quality and overall process economics [35,68].

#### 1.4.4. Publication Trends and Growth Dynamics

A temporal analysis of publication frequency reveals exponential growth in research on the valorisation of lemon waste, particularly post 2015. This acceleration coincides with increased policy emphasis on circular economy (EU Circular Economy Action Plan, 2015) and growing industrial interest in bio-based products [69]. The period 2019–2025 accounts for approximately 58% of total publications, indicating that this field remains in active expansion rather than maturity. This growth trajectory mirrors broader trends in food-waste valorisation research, which has experienced a four-fold increase in annual publications since 2010 [70].

Geographically, the scientific literature on citrus-waste valorisation is intensely concentrated in citrus-producing regions around the Mediterranean, particularly Italy and Spain, where numerous case studies and process developments have been reported [71].

In parallel, substantial contributions also originate from prominent citrus-producing emerging economies such as Brazil, India, and China, reflecting both the abundance of feedstock and growing policy interest in circular-bioeconomy strategies for agricultural residues [71].

North American groups, especially in the United States, remain active in citrus-derived bioprocessing and biorefinery research despite the decline of some domestic citrus sectors, often placing greater emphasis on process intensification, biofuels, and biotransformations than on regional feedstock availability [72].

#### 1.4.5. Journal Distribution and Disciplinary Scope

Publications span diverse disciplinary domains, with predominant representation in food science and technology journals (e.g., *Food Chemistry*, *Journal of Food Science and Technology*, *LWT—Food Science and Technology*), followed by environmental- and sustainability-focused outlets (*Journal of Cleaner Production*, *Waste Management*, *Bioresource Technology*), and chemical engineering publications (*Industrial Crops and Products*, *Journal of Chemical Technology and Biotechnology*). This multidisciplinary approach reflects the inherently integrative nature of biorefinery research, which requires expertise spanning chemistry, engineering, biology, and sustainability science.

Interestingly, relatively few publications appear in high-impact, broad-scope journals (e.g., *Nature*, *Science*, *PNAS)*, *suggesting that citrus-waste valorisation*, *despite its practical importance*, *has not yet achieved the* scientific visibility of other biorefinery feedstocks (e.g., lignocellulosic biomass, algae). This may reflect the perception of citrus valorisation as an applied, incremental field rather than one that yields transformative scientific insights. However, recent advances in nanocellulose production from citrus waste and in novel hydrodynamic cavitation extraction methods signal potential for heightened scientific interest [73,74].

### 1.5. Research Trends and Knowledge Gaps

The bibliometric analysis reveals a vibrant research landscape characterised by sustained growth, methodological innovation, and expanding product portfolios. However, it also exposes critical knowledge gaps and imbalanced research priorities that must be addressed to enable commercial-scale implementation of lemon-biorefinery concepts. These gaps, systematically identified through network analysis and literature synthesis, provide a roadmap for future research directions.

#### Identified Research Gaps

Bibliometric analysis and systematic literature review reveal eight critical research gaps that constrain the translation of lemon-biorefinery concepts from laboratory investigations to industrial implementation (Table 1). These gaps span technical, economic, environmental, and market-development dimensions, collectively highlighting the need for more integrated, application-oriented, and scale-conscious research approaches. Addressing these deficiencies is essential to realising the circular-bioeconomy potential of lemon-processing residues.

The environmental imperative for citrus-waste valorisation extends beyond greenhouse gas reduction and landfill diversion to encompass preventing water pollution and protecting biodiversity. Unprocessed citrus waste contains high concentrations of D-limonene and other monoterpenes, which exhibit phytotoxicity and antimicrobial activity, rendering the waste unsuitable for direct agricultural application whilst causing aquatic toxicity when leachate enters waterways. Green extraction technologies simultaneously recover these valuable compounds for commercial applications whilst mitigating environmental risks [66].

## 2. Lemon Composition and Residue Characterisation

Understanding the chemical composition and quantitative distribution of lemon-processing residues is fundamental to designing efficient cascade-valorisation strategies. Lemon fruit *(Citrus × limon*) comprises distinct anatomical fractions, each characterised by unique biochemical profiles that determine their suitability for specific valorisation pathways. This section provides a comprehensive overview of the compositional characteristics of lemon residues, typical yields from industrial processing, and the variability introduced by cultivar selection and geographical factors.

### 2.1. Chemical Composition of Lemon Fractions

Lemon-fruit processing for juice extraction generates substantial residues comprising peel (flavedo and albedo), seeds, and pomace, collectively representing 45–55% of total fruit weight [16]. The chemical composition of these fractions exhibits considerable heterogeneity, reflecting their distinct physiological functions and cellular structures (Table 2).

#### 2.1.1. Flavedo (External Peel)

The flavedo, or epicarp, is the outermost pigmented layer of the lemon peel, typically 1–3 mm thick and accounting for approximately 8–10% of total fruit weight [32,94,95]. This tissue is characterised by the presence of oil glands containing essential oils, chromoplasts responsible for colour, and epidermal cells with characteristic cuticle structures. The flavedo serves as the primary reservoir for volatile compounds and lipophilic bioactive constituents [32].

Compositionally, the flavedo is distinguished by high concentrations of essential oils (2.0–4.5% on a dry-weight basis), with D-limonene comprising 60–76% of the volatile fraction depending on cultivar and maturity stage [96,97]. Other significant volatile components include β-pinene (8–12%), γ-terpinene (6–10%), and α-pinene (1–3%). The flavedo also contains elevated levels of polymethoxylated flavones (PMFs), particularly tangeretin and sinensetin, which are virtually absent in the albedo [85,93]. Total phenolic content in flavedo ranges from 102–139 mg galacturonic acid equivalents (GAE) per gram of dry weight, significantly exceeding albedo concentrations [85].

The structural polysaccharide content of flavedo is relatively limited compared to albedo, with cellulose (8–12%), hemicellulose (4–7%), and pectin (12–18%) present in lower concentrations. The moisture content of fresh flavedo typically ranges from 70–76% [85].

#### 2.1.2. Albedo (Internal Peel)

The albedo, or mesocarp, comprises the white spongy tissue beneath the flavedo, constituting the bulk of lemon-peel mass (25–35% of total fruit weight). This tissue exhibits a highly porous structure composed predominantly of parenchyma cells interspersed with vascular bundles, conferring exceptional water-holding and oil-binding capacities [85].

The albedo is characterised by high concentrations of structural polysaccharides, particularly pectin (18–28% dry weight), cellulose (15–22%), and hemicellulose (8–14%) [85]. Pectin isolated from lemon albedo exhibits high methoxyl content (degree of esterification 55–75%), rendering it suitable for gel formation under acidic conditions [30]. The albedo contains a substantially lower essential-oil content (<0.5%) than flavedo, but it serves as the primary source of dietary fibre in lemon-processing residues.

Phenolic compounds in albedo, whilst less concentrated than in flavedo (84–120 mg GAE per gram of dry weight), comprise valuable flavanone glycosides including hesperidin and eriocitrin, which exhibit significant bioactivity [85,87]. The moisture content of fresh albedo ranges from 65–70%, with an ash content of 3.5–5.0% dry weight reflecting mineral composition [85].

#### 2.1.3. Seeds

Lemon seeds, though representing a minor fraction by mass (1–5% of whole fruit, depending on cultivar and fruit size), constitute a valuable source of oil and protein [89]. Individual seed weight ranges from 0.08 to 0.15 g, and typical lemons contain 5–15 seeds, depending on variety and pollination conditions.

Lemon seeds contain 27–45% extractable oil on a dry-weight basis, with yields varying according to extraction method and seed maturity [34,89,98]. The fatty acid profile of lemon-seed oil is dominated by unsaturated fatty acids, particularly linoleic acid (C18:2, 34–42%), oleic acid (C18:1, 24–32%), and palmitic acid (C16:0, 18–24%) [99,100].

Protein content in defatted lemon-seed meal ranges between 8 and 15% dry weight, with fibre content of 5–9% and ash content of 4–6% [100]. Lemon seeds contain bioactive limonoids and phenolic compounds, including naringin and hesperidin, whose concentrations vary widely depending on extraction method and cultivar, typically ranging from 0.08 to 80.9 mg per gram of dry weight [101,102]. The moisture content of fresh seeds generally ranges from 45 to 55% before drying [103].

#### 2.1.4. Pomace (Pulp Residue)

Lemon pomace refers to the residual pressed pulp obtained after juice extraction, composed predominantly of juice vesicle membranes, segment walls (endocarp), and fibrous rag [87,104,105]. This solid fraction generally accounts for approximately 15–25% of the original fresh-fruit weight, with slight variation depending on the efficiency of the juice-extraction process [103,106,107].

Pomace composition is characterised by moderate levels of structural carbohydrates, including cellulose (12–18%), hemicellulose (6–10%), and residual pectin (8–15%), along with retained soluble sugars (glucose, fructose, sucrose) at 5–12% dry weight [91]. The pomace fraction contains significant concentrations of organic acids, particularly citric acid (8–15% dry weight), making it a potential feedstock for citric acid recovery [92].

The phenolic content of lemon pomace is intermediate between that of peel and seed fractions, generally ranging from 10 to 30 mg GAE per gram of dry weight under conventional extraction conditions, and potentially reaching up to 45 mg GAE per gram of dry weight when optimised extraction methods such as NADES are employed [102,108,109]. Hesperidin represents the predominant flavanone within this matrix [110]. The moisture content of fresh pomace is high—typically between 75 and 85%—necessitating dewatering before storage or further processing [109]. The protein fraction usually ranges from 4 to 8% dry weight, while the ash content remains between 3 and 5% dry weight [111,112].

The compositional variability observed across lemon fractions is further compounded by cultivar-specific differences, which significantly influence the concentration of extractable bioactive compounds. Commercial lemon production worldwide relies predominantly on a limited number of cultivars, each exhibiting distinct phytochemical profiles that directly impact biorefinery design and process optimisation. Table 3 presents a comparative overview of key bioactive-compound concentrations across four major commercial lemon cultivars, highlighting the substantial variability in essential-oil composition, pectin yield, and hesperidin content that must be considered when developing standardised extraction protocols.

The data presented in Table 3 reveal considerable inter-cultivar variability, with Femminello demonstrating the highest concentrations across all three parameters evaluated—limonene content (68–75%), pectin yield (24.0–29.0%), and hesperidin concentration (22.0–35.0 mg·g^−1^)—whilst Verna exhibits comparatively lower values. These differences have direct implications for biorefinery economics: feedstock selection based on cultivar availability may necessitate process adjustments to maintain consistent product specifications. Furthermore, the observed variability underscores the importance of characterising regional lemon supplies before industrial-scale implementation, as compositional differences of this magnitude can substantially affect extraction efficiency and product quality.

### 2.2. Quantification of Processing Residues

The mass distribution of lemon fractions after juice processing is influenced by fruit morphology, extraction technology, and cultivar-specific characteristics [119,120].

**Figure 5:**
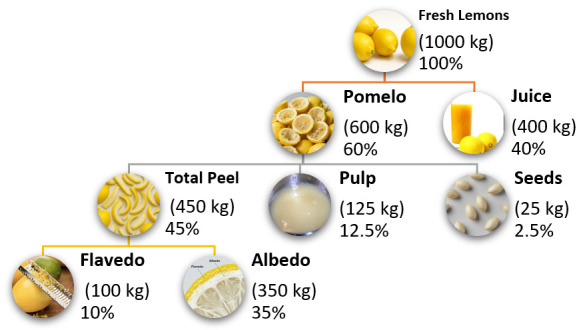
Typical mass balance of lemon-processing residues at industrial scale. Typical yields of residue fractions (peel, pulp, seeds) obtained per tonne of fresh lemons processed, illustrating the quantitative distribution of waste streams available for cascading-valorisation strategies.

Figure 5 illustrates typical yields of residue fractions obtained per tonne of fresh lemons processed under industrial-scale operations. In a representative processing scenario, 1000 kg of whole lemons generally yield about 350–450 kg of juice—corresponding to an extraction efficiency of 35–45%—with the remaining 550–650 kg corresponding to solid residues (peel, pulp, seeds, and rag) [119,121].

These yields represent average values for commercial processing operations employing mechanical extraction systems. The adoption of green technologies may alter residue distribution, typically increasing juice yield by 5–10% while proportionally reducing pomace generation [47,122,123].

The dry-matter content of residue fractions significantly impacts handling, storage, and subsequent processing requirements [124]. On a dry-weight basis, processing 1000 kg of fresh lemons yields approximately 150–200 kg of total dry residue, distributed as follows:

• Dried peel (combined): 100–130 kg [125]
• Dried seeds: 5–25 kg [123,125]
• Dried pomace: 15–35 kg [123]
• Soluble solids lost to wastewater: 5–15 kg [123,124]

These quantities establish the feedstock availability for cascade-valorisation operations and serve as key parameters for techno-economic assessments of biorefinery viability [126,127].

## 3. The Hierarchy of Value-Added Products

The intrinsic diversity of lemon residues offers a rich palette of value-added compounds, each possessing distinct physicochemical properties and economic appeal [128]. Cascade valorisation establishes a logical sequence for their recovery, prioritising the following:

• **Essential Oils and Volatiles**: The initial fractionation stage typically involves cold pressing or hydrodistillation to recover essential oils, highly prized by the food, flavour, and cosmetic industries. These comprise monoterpenes such as limonene and alpha-terpineol, as well as bioactive sesquiterpenes with antioxidant, antimicrobial, and therapeutic applications [61,129,130,131,132,133].
• **Pectin**: Next, peels and rag residues undergo acid- or enzyme-assisted extraction to yield pectin, a functional polysaccharide used as a gelling agent, stabiliser, and dietary fibre. Cascade valorisation enhances pectin’s techno-economic feasibility by integrating extraction with upstream oil separation and downstream polyphenol recovery [21,30,31,134,135].
• **Polyphenols and Flavonoids**: Targeted extraction of polyphenols—including flavanones, flavones, and flavonols such as hesperidin and naringin—draws on solvent and enzymatic processes optimised for yield, purity, and functional value within the nutraceutical and pharmaceutical sectors [109,136,137,138,139].
• **Cellulose and Nanocellulose**: Post pectin extraction, the remaining lemon biomass, which is notably rich in cellulose and hemicellulose, can be processed using green mechanical or chemical pretreatments to obtain microcrystalline cellulose, nanocellulose crystals (NCC), and nanofibrils (NFC). These materials exhibit exceptional mechanical, rheological, and barrier properties, making them valuable for advanced applications in biopolymer composites, pharmaceuticals, and functional foods [67,74,125,140].
• **Lignocellulosic Biomass Valorisation**: Following the removal of limonene, pectin, polyphenols, and cellulose derivatives, the residual solid matrix—composed mainly of cellulose, hemicellulose, and lignin—is well suited for biotechnological upgrading. Solid-state fermentation (SSF) enables the use of specialised fungi (e.g., *Aspergillus niger*) and yeasts (e.g., *Saccharomyces cerevisiae*) to produce industrially relevant enzymes (e.g., cellulases, xylanases) and single-cell protein (SCP) for food, feed, or biocatalytic applications [141,142,143,144,145].
• **Bioenergy, Biochar, and Soil Amendments**: The final valorisation step transforms recalcitrant residues (pomace, seeds, effluent solids) through anaerobic digestion [146], pyrolysis [147,148], and composting [149], providing bioethanol [54], biohydrogen [150], and biofertilisers [151] that closes the resource recovery loop.

The hierarchical cascade framework described above generates multiple product streams from sequential processing of lemon residue fractions. Each fraction—essential oils, peel and rag, seeds and pomace, aqueous effluents, and recalcitrant residues—yields distinct bioproducts through specific extraction or biotransformation pathways. Table 4 summarises the principal products, extraction methodologies, and industrial applications associated with each fraction, providing a consolidated overview of the complete valorisation landscape within integrated lemon-biorefinery systems.

As illustrated in Table 4, the cascade-valorisation approach enables comprehensive utilisation of lemon-processing residues, transforming what would otherwise constitute disposal liabilities into diversified revenue streams spanning the food, pharmaceutical, cosmetic, and bioenergy sectors. The progression from high-value volatile compounds through structural polysaccharides to residual biomass for energy recovery exemplifies the biorefinery principle of maximising value extraction whilst minimising waste generation. Critically, the sequential nature of these pathways requires careful process integration to ensure that upstream extraction does not compromise downstream product quality or yield.

## 4. Primary Valorisation Pathways

Primary valorisation pathways for lemon-processing residues encompass the recovery of essential oils, pectin, seed oils, and citric acid through conventional and emerging green extraction technologies. These pathways constitute the foundation of cascade-biorefinery systems, targeting compounds that are either readily extractable or present in substantial concentrations within specific lemon fractions.

The selection of extraction methodology critically influences product yield, purity, economic viability, and environmental sustainability. Conventional approaches, including cold pressing, hydrodistillation, and acid extraction, have established industrial precedents and regulatory acceptance but suffer from long processing times, high energy consumption, and limited selectivity.

In contrast, green extraction technologies—microwave-assisted extraction (MAE), ultrasound-assisted extraction (UAE), supercritical fluid extraction (SC-CO_2_), and enzyme-assisted extraction (EAE)—offer substantial improvements across multiple performance dimensions, including yield enhancement (16–112% over conventional methods), processing-time reduction (89–98%), and energy-efficiency gains (up to 95% reduction).

Table 5 synthesises the key characteristics, optimal conditions, typical yields, and industrial applications of primary valorisation pathways, providing a comparative framework for technology selection and process design in implementing a lemon biorefinery.

The comparative synthesis presented in Table 5 underscores a paradigm shift in citrus-waste valorisation, in which conventional extraction methodologies are being progressively supplanted by green technologies that offer superior performance across yield, energy efficiency, environmental impact, and product quality.

Microwave-assisted and ultrasound-assisted extractions are particularly promising for industrial implementation, combining substantial yield improvements (16–50%) with dramatic reductions in processing time (89–98%) and moderate capital investment requirements.

Supercritical CO_2_ extraction, whilst demanding higher capital expenditure, uniquely delivers pharmaceutical-grade purity and complete solvent elimination, justifying deployment for high-value applications where premium pricing offsets equipment costs. Enzyme-assisted extraction provides unmatched selectivity and biotransformation capability, converting glycosides into bioavailable aglycones, and recent advances in low-cost fungal enzyme production have substantially improved the economic feasibility.

Critically, optimal biorefinery design necessitates sequential integration of complementary technologies rather than reliance on single extraction methods: supercritical CO_2_ or solvent-free microwave extraction for initial essential-oil recovery, followed by ultrasound-assisted or enzyme-assisted polyphenol extraction, microwave-assisted pectin isolation, and concluding with fermentation-based citric acid production or anaerobic digestion of residual biomass. This cascading approach maximises cumulative value recovery, transforming disposal costs into diversified revenue streams whilst advancing circular-economy principles through comprehensive resource utilisation.

The successful translation of these laboratory- and pilot-scale advances to commercial implementation remains contingent upon addressing scale-up engineering challenges, establishing stable markets for multiple product streams, and securing favourable regulatory approval for novel extraction processes and resultant ingredients—challenges that Part II of this review series systematically examines.

## 5. Advanced Valorisation Frontiers

The cascade-biorefinery concept extends beyond primary products to encompass advanced, high-value materials that command premium market positions and enable diversified revenue streams. Whilst essential oils, pectin, seed oils, and citric acid represent established valorisation pathways, the extraction of bioactive compounds, the production of industrial enzymes, the isolation of α-cellulose, and the synthesis of nanocrystalline cellulose constitute frontier technologies offering substantially enhanced economic returns. These advanced products align with emerging market demand for functional biomaterials, pharmaceutical intermediates, and sustainable nanomaterials, thereby positioning lemon biorefineries at the intersection of circular-economy principles and high-tech applications.

### 5.1. Bioactive Compounds and Antioxidants

Beyond the primary metabolites targeted by conventional extraction processes, lemon-processing residues harbour a diverse array of bioactive phytochemicals with potent antioxidant, antimicrobial, anti-inflammatory, and nutraceutical properties. The strategic isolation and purification of these compounds transform lemon waste from a disposal liability into a source of high-value pharmaceutical and nutraceutical ingredients, with applications spanning functional foods, cosmeceuticals, and therapeutic formulations [177,178].

#### 5.1.1. Polyphenolic Composition and Antioxidant Activity

Lemon peels, particularly the flavedo and albedo fractions, contain substantial concentrations of flavonoids, polymethoxylated flavones (PMFs), and phenolic acids that collectively contribute to exceptional antioxidant capacity. The predominant flavanone glycoside in lemon is eriocitrin (eriodictyol-7-O-rutinoside), which exhibits superior bioavailability relative to hesperidin from oranges due to its enhanced aqueous solubility. Eriocitrin content in lemon-peel extracts ranges from 3.5 to 7.2 mg per gram of dry weight, depending on extraction methodology and cultivar characteristics [179].

Total phenolic content (TPC) in lemon-peel extracts varies considerably, ranging from 84 to 139 mg gallic acid equivalents (GAE) per gram of dry weight in the albedo and 102 to 139 mg GAE per gram of dry weight in the flavedo. This compositional heterogeneity reflects the different distribution of flavonoids between peel layers, with polymethoxylated flavones—including tangeretin, sinensetin, and nobiletin—primarily concentrated in the outer flavedo layer. These PMFs, although present at lower absolute concentrations than flavanones, exhibit potent bioactivities, including anti-inflammatory, neuroprotective, and anticarcinogenic effects [180].

The antioxidant capacity of lemon-peel extracts, as assessed by complementary in vitro assays, demonstrates substantial free-radical-scavenging efficacy [30,33,181]. Lemon essential oil exhibited the highest 2,2-diphenyl-1-picrylhydrazyl (DPPH) free-radical-scavenging activity and cupric-ion-reducing antioxidant capacity (CUPRAC) amongst citrus species in comparative studies, whilst mandarin oil showed superior 2,2′-azino-bis3-ethylbenzothiazoline-6-sulphonic acid (ABTS) radical-cation reduction and ferric-reducing antioxidant power (FRAP) [178]. This dichotomy in antioxidant assay performance reflects the complex interplay between phenolic structure and reaction-mechanism specificity, underscoring the importance of employing multiple complementary assay methods for comprehensive antioxidant characterisation.

#### 5.1.2. Advanced Extraction Technologies for Bioactive Recovery

The efficiency and selectivity of bioactive-compound extraction depend critically on the selection and optimisation of extraction methodologies. Recent technological advances have emphasised green extraction approaches that minimise solvent consumption, reduce energy requirements, and preserve the structural integrity of thermolabile bioactive molecules [182].

Pulsed electric field (PEF)-assisted extraction represents a non-thermal processing technology employing short-duration, high-voltage pulses (10–80 kV·cm^−1^) to induce membrane permeabilisation through electroporation, thereby facilitating the release of intracellular bioactive compounds [170]. The application of PEF pretreatment to lemon peel significantly enhanced eriocitrin recovery, achieving concentrations of 7.2 ± 0.2 mg per gram of dry weight under optimised conditions combining PEF with hydroethanolic extraction [183]. The enhancement mechanism involves the formation of aqueous pores in cellular membranes, thereby increasing tissue permeability and reducing mass-transfer resistance during subsequent solvent extraction.

Ultrasound-assisted extraction (UAE) employs acoustic cavitation to disrupt cellular structures and enhance mass-transfer kinetics. Comparative studies have demonstrated that UAE reduces extraction time from 185 min (conventional Soxhlet extraction) to 20 min whilst maintaining or improving bioactive-compound yields [184]. The combination of ultrasound and hydroethanolic solvents yielded a total phenolic content ranging from 102 to 139 mg GAE per gram of dry weight from lemon peels, with ascorbic acid concentrations reaching 85–120 mg per 100 g of fresh weight [184].

Microwave-assisted extraction (MAE) utilises selective dielectric heating to rapidly elevate intracellular temperatures, causing cellular disruption through the generation of internal vapour pressure. This technology demonstrates efficacy for polar bioactive compounds, achieving extraction efficiencies comparable to or exceeding conventional methods whilst consuming substantially less energy and time [130]. The combination of MAE with citric acid–glycerol deep eutectic solvents (DES) has emerged as an innovative green extraction strategy, simultaneously recovering pectin, micro-cellulose, and polyphenolic extracts in a sequential, integrated process [185].

#### 5.1.3. Bioactive Applications and Market Potential

The extracted bioactive compounds from lemon waste demonstrate substantial commercial potential across multiple industrial sectors. In the functional-food industry, standardised lemon flavonoid extracts serve as natural antioxidants replacing synthetic alternatives such as butylated hydroxyanisole (BHA) and butylated hydroxytoluene (BHT), addressing consumer demand for “clean-label” formulations [164]. The incorporation of lemon-peel extracts into active packaging systems has demonstrated efficacy in extending the shelf life of fresh produce through antioxidant and antimicrobial activity, reducing malonaldehyde (MDA) formation in meat products during refrigerated storage [39].

In the nutraceutical sector, eriocitrin-rich lemon extracts exhibit superior bioavailability compared to hesperidin-rich orange extracts in human pharmacokinetic studies, with plasma concentrations reaching higher levels following equivalent flavanone dosing [186,187]. This enhanced bioavailability positions lemon bioactive extracts as preferential sources for cardiovascular health supplements, given the documented vasodilatory, anti-hypertensive, and lipid-modulating properties of citrus flavanones [117,188].

Lemon-peel extracts demonstrate efficacy in reducing oxidative stress markers in dermal fibroblasts, suggesting applications in topical formulations targeting skin senescence and photodamage [182].

The transformation of lemon-processing waste into standardised bioactive extracts thus addresses both environmental sustainability imperatives and commercial market opportunities, exemplifying the economic viability of advanced biorefinery concepts.

### 5.2. Industrial Enzymes Production

It is essential to distinguish the biotechnological production of industrial enzymes—addressed in this section—from enzyme-assisted extraction (EAE), which is examined in Section 7.4. These represent fundamentally different valorisation strategies with distinct objectives and mechanisms. In enzyme production, lemon-processing residues serve as fermentation substrates for microbial biosynthesis, wherein fungi or bacteria convert the carbohydrate-rich waste matrix into new high-value biocatalysts (pectinases, cellulases, xylanases) that did not previously exist in the feedstock. This constitutes a biotransformation pathway that generates novel products through microbial metabolism. Conversely, enzyme-assisted extraction employs commercially available or in-house-produced enzymes as processing aids to facilitate the release of pre-existing bioactive compounds from plant cell walls, functioning as a recovery technology analogous to ultrasound- or microwave-assisted methods. Understanding this conceptual distinction is critical for biorefinery design, as enzyme production constitutes an independent revenue stream, whilst EAE constitutes a process-intensification strategy for enhancing the recovery of other target compounds.

The enzymatic valorisation of lemon-processing residues presents a dual opportunity: citrus waste serves both as an inexpensive substrate for enzyme production via fermentation and as a source of endogenous plant enzymes that can be recovered and utilised industrially. The production of hydrolytic enzymes, particularly pectinases, cellulases, xylanases, and ligninases—from citrus waste via solid-state or submerged fermentation—aligns perfectly with circular-bioeconomy principles, converting a low-value by-product into high-value biocatalysts with diverse industrial applications [141].

#### Microbial-Enzyme Production from Citrus Waste

Citrus-processing waste, composed of readily fermentable polysaccharides (pectin, cellulose, hemicellulose) and residual sugars, constitutes an excellent substrate for microbial-enzyme production. The global enzyme market was valued at USD 60.48 billion in 2023 and is projected to grow at a compound annual growth rate of 6.5% through 2030, driven by increasing demand in the food-processing, textiles, pharmaceuticals, and biofuel industries [189].

Solid-state fermentation (SSF) employing citrus peels as a substrate has demonstrated efficacy for pectinase production. For *Aspergillus niger*, *Aspergillus oryzae*, and *Penicillium* spp. cultivated on citrus waste, the polygalacturonase activities ranged from 1600 to 1700 U per gram of substrate after 36 h of cultivation, representing 25% higher yields compared to apple pomace substrates under identical fermentation conditions [190]. The high pectin content of citrus peels (18–28% dry weight) serves as an effective inducer for pectinase enzyme systems, obviating the need for expensive commercial pectin supplements in fermentation media.

Cellulase and xylanase production from citrus waste has similarly been demonstrated across multiple fungal species. Recent investigations documented that lemon peel exhibited the highest cellulase-specific activity (4481 U per milligram of protein) amongst diverse fruit peels evaluated under anaerobic fermentation conditions, followed by orange and tangerine peels [191]. Concurrent production of multiple enzyme activities, including cellulase, xylanase, and pectinase, from single fermentation processes represents an economically attractive bioprocessing strategy, reducing production costs through substrate sharing and process integration [192].

### 5.3. Cellulose and Nanocellulose Production

Following sequential extraction of essential oils, pectin, and bioactive compounds, the remaining lemon-processing residues retain substantial quantities of structural polysaccharides, predominantly cellulose, hemicellulose, and residual lignin. The selective isolation of α-cellulose—the high-purity, high-molecular-weight cellulose fraction—from these residues provides access to a versatile biomaterial platform suitable for subsequent conversion into dissolving pulp, cellulose derivatives, and advanced nanomaterials [35,67,74]. The inherently low lignin content of citrus biomass (2–3.5% in fresh peels) simplifies delignification processes compared to wood-derived feedstocks, reducing both chemical consumption and processing severity [61].

**Figure 6:**
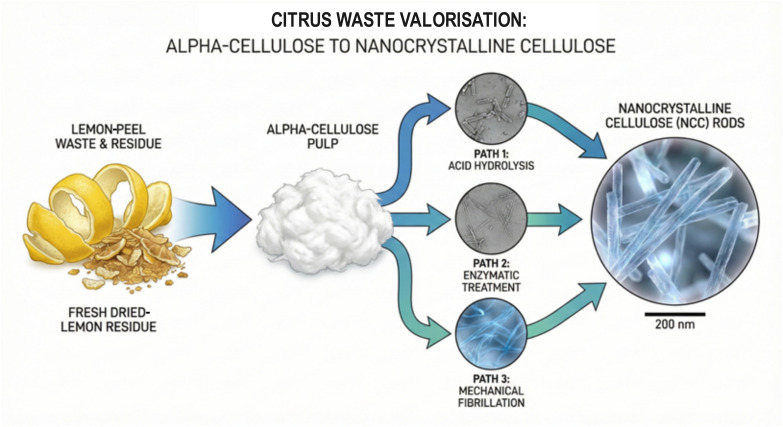
Schematic representation of citrus-waste valorisation pathways to produce nanocrystalline cellulose (NCC). Transmission electron microscopy images illustrate the characteristic rod-like morphology of the resulting nanocrystals (scale bar: 200 nm).

Figure 6 illustrates the principal valorisation pathways from lemon-peel waste through α-cellulose pulp to nanocrystalline cellulose (NCC). Fresh and dried lemon-peel residues are processed to obtain α-cellulose pulp, which is subsequently converted to NCC rods through three principal routes: acid hydrolysis (Path 1), enzymatic treatment (Path 2), and mechanical fibrillation (Path 3).

The production of cellulosic materials from lemon-processing waste encompasses both the isolation of α-cellulose and its subsequent transformation into nanocrystalline cellulose. Table 6 summarises the principal methodologies employed for cellulosic material synthesis, comparing conventional alkaline/oxidative treatments with emerging green technologies and various NCC production routes. The selection of an appropriate method depends upon the desired product characteristics, including purity, crystallinity, surface chemistry, and intended application.

Conventional α-cellulose isolation employs sequential alkaline hydrolysis with sodium hydroxide solutions (2–17.5% *w*/*v*) at 60–100 °C, followed by oxidative bleaching with hydrogen peroxide or sodium hypochlorite to remove residual lignin and chromophoric compounds [193,199]. Chlorine-free bleaching sequences employing hydrogen peroxide under alkaline conditions represent environmentally preferable alternatives, achieving α-cellulose purities exceeding 85% [193].

Hydrodynamic cavitation has emerged as a green alternative for cellulose extraction, generating localised high-pressure and high-temperature conditions through controlled bubble collapse without requiring chemical additives [74,194]. This technology, demonstrated at a semi-industrial scale, produces micronised cellulose (“CytroCell”) with distinctive properties including low crystallinity (45–55%), high porosity, and good water-holding capacity [194].

For the production of nanocrystalline cellulose (NCC), sulphuric acid hydrolysis remains the most widely used method, yielding NCC with dimensions of 8–12 nm in width and 150–250 nm in length and a crystallinity index of approximately 71% [125,195,196]. Ammonium persulphate oxidation produces NCC with the highest reported crystallinity index (73.8%) amongst citrus-derived materials, whilst TEMPO-mediated oxidation achieves the highest yields (~19%) under mild conditions that preserve surface hydroxyl groups for subsequent functionalisation [197,198].

The exceptional properties of nanocrystalline cellulose—including nanoscale dimensions (3–50 nm width, 50–500 nm length), high tensile strength (~7500 MPa), crystallinity exceeding 70%, and large specific surface area (~150 m^2^·g^−1^)—enable diverse high-value applications across multiple industrial sectors [35].

Table 7 presents the principal application fields of lemon-derived NCC and correlates functional properties with specific mechanisms of action.

In nanocomposite applications, NCC serves as a mechanical reinforcing agent, with percolation network formation at loadings as low as 3–5 wt%, significantly enhancing composite tensile strength and elastic modulus through stress transfer mechanisms [201]. Citrus-derived NCC demonstrates reinforcement efficacy comparable to wood-derived NCC in polylactic acid, polyvinyl alcohol, and starch-based biocomposites [200].

Cellulose nanocrystals and nanofibrils extracted from lemon seeds have been successfully used to stabilise oil-in-water Pickering emulsions with long-term physical stability, without the need for synthetic surfactants, highlighting their potential for food, cosmetic, and pharmaceutical formulations [208].

The commercial deployment of citrus-derived NCC faces several technical and economic challenges, including energy-intensive downstream processing, yield-optimisation requirements, and scale-up limitations [209]. Current NCC pricing (USD 50–200 per kg for research-grade material) restricts commercialisation to high-value speciality applications [210]. However, techno-economic modelling demonstrates that when NCC production is integrated within cascade-biorefinery frameworks—sequentially extracting essential oils, pectin, and polyphenols before cellulose isolation—the cumulative value recovery substantially exceeds processing costs [71]. Life-cycle assessment studies further indicate that citrus-derived NCC exhibits a favourable environmental performance compared with wood-derived alternatives, primarily due to the avoidance of energy-intensive delignification processes required by the high lignin content of woody biomass [201,211]. The optimal positioning of NCC production within the lemon cascade-biorefinery framework thus maximises both economic viability and environmental sustainability, transforming cellulosic residues into advanced nanomaterials whilst contributing to comprehensive biomass utilisation.

## 6. Biotechnological Valorisation: Microbial Bioconversion and Bioenergy Production

Whilst the preceding sections have addressed the recovery of pre-existing compounds via extraction technologies and the isolation of structural polysaccharides, this section examines biotechnological pathways in which microorganisms convert lemon-processing residues into novel products via metabolic conversion. These bioconversion strategies extend beyond compound recovery to encompass the biosynthesis of microbial biomass for food and feed applications, the fermentative production of biofuels and platform chemicals, and the biological stabilisation of residual organic matter into soil amendments. Such transformations position lemon waste as a renewable feedstock for the bioeconomy, complementing extraction-based valorisation within integrated cascade-biorefinery frameworks.

Table 8 provides a comprehensive synthesis of biotechnological valorisation pathways for lemon-processing residues, encompassing microbial bioconversion for nutritional applications, bioenergy production through anaerobic and fermentative processes, platform-chemical biosynthesis, and biological-waste stabilisation.

### 6.1. Microbial-Biomass Production for Food and Feed Applications

Beyond enzyme biosynthesis (Section 5.2), lemon-processing residues serve as substrates for the cultivation of microbial biomass intended for direct nutritional applications in animal feed and, prospectively, human food systems [141,145]. The fermentable carbohydrate content of citrus waste—comprising glucose, fructose, sucrose, and hydrolysable polysaccharides—supports vigorous microbial growth, enabling the bioconversion of low-value sugars into protein-rich cellular biomass.

Single-cell protein (SCP) production employs yeasts (*Saccharomyces cerevisiae*, *Candida utilis*) or filamentous fungi (*Aspergillus oryzae*, *Rhizopus oligosporus*) cultivated on citrus hydrolysates or solid residues [141]. Yeast cultivation on citrus-peel hydrolysates yields 0.4–0.5 g of biomass per gram of consumed sugar, with a crude protein content of 45–55% and a favourable essential amino acid profile suitable for aquaculture and monogastric animal feeds [145]. Solid-state fermentation with filamentous fungi enriches the protein content of citrus residues by 18–25% whilst simultaneously improving digestibility through partial degradation of structural polysaccharides and reduction in anti-nutritional factors [141,143].

Ganatsios et al. [145] demonstrated the innovative valorisation of orange-peel waste through yeast cultivation, producing brewing biocatalysts capable of generating aromatic beer whilst simultaneously yielding yeast extract as an alternative to commercial preparations. This dual-product approach exemplifies the biorefinery principle of maximising value recovery through integrated bioconversion strategies.

The cultivation of probiotic microorganisms on citrus substrates is an emerging area, with *Lactobacillus* spp. achieving viable counts of 10^9^–10^10^ colony-forming units per millilitre when cultivated on a citrus-pomace medium [145]. The residual dietary fibre and polyphenolic compounds in citrus substrates may confer prebiotic properties, potentially enhancing the functional value of probiotic preparations derived from these waste streams.

### 6.2. Bioenergy Production: Bioethanol, Biomethane, and Biohydrogen

The conversion of lemon-processing residues into bioenergy carriers addresses both waste-management imperatives and renewable-energy targets, transforming disposal liabilities into sustainable fuel sources [54,61,72]. Three principal bioenergy pathways merit consideration: ethanolic fermentation of hydrolysed sugars, anaerobic digestion for biomethane production, and dark fermentation for biohydrogen generation.

Bioethanol production from citrus waste requires preliminary detoxification to remove D-limonene, which inhibits *Saccharomyces cerevisiae* at concentrations exceeding 0.5% (*v*/*v*) [54,72]. Teke et al. [54] developed an integrated approach in which ultrasound-assisted extraction first recovers D-limonene (32.9 mg·g^−1^ dry weight) as a valuable co-product, thereby enabling fermentation of detoxified hydrolysates with ethanol yields of 35–45 L per tonne of fresh citrus-peel waste. Thermotolerant yeasts, such as *Kluyveromyces marxianus*, offer advantages for simultaneous saccharification and fermentation at elevated temperatures (37–42 °C), thereby reducing cooling requirements and the risk of contamination [72].

**Anaerobic digestion** of citrus residues generates biomethane suitable for combined heat and power generation or grid injection following upgrading [61,146]. The antimicrobial properties of D-limonene present a critical operational challenge, inhibiting methanogenic archaea at concentrations exceeding 400 mg·L^−1^ [63,81]. Calabrò et al. [81] systematically investigated the effect of essential-oil concentration on orange-peel biomethanisation, establishing that D-limonene removal or dilution below inhibitory thresholds is necessary for stable digester operation. Delimonened citrus waste yields 450–550 mL of methane per gram of volatile solids, with biogas containing 60–70% methane [61,146]. Co-digestion with nitrogen-rich substrates (e.g., sewage sludge, manure) improves C:N ratios and buffering capacity, thereby enhancing process stability [146]. Jiang et al. [62] demonstrated that the addition of citrus-peel biochar to anaerobic digesters promotes direct interspecies electron transfer, accelerating methanogenesis and increasing methane yields by 15–25%.

Biohydrogen production via dark fermentation of citrus-processing effluents represents a nascent yet promising pathway [150,159]. Torquato et al. [150] achieved hydrogen yields of 85–120 mL per gram of chemical oxygen demand from citrus-processing wastewater using anaerobic bacteria enriched from sewage sludge, with hydrogen purities exceeding 90%. The integration of dark fermentation with subsequent methanogenic stages (two-stage anaerobic digestion) maximises energy recovery whilst producing both hydrogen and methane from sequential process streams [159].

### 6.3. Platform-Chemical Biosynthesis and Biological Stabilisation

Beyond direct bioenergy applications, fermentative processes enable the biosynthesis of platform chemicals serving as precursors for bioplastics, adhesives, and speciality chemicals [61,126].

Volatile fatty acid (VFA) production through acidogenic fermentation of citrus hydrolysates yields mixtures of acetate, propionate, and butyrate at concentrations of 0.3–0.5 g per gram of volatile solids [126]. These VFAs serve as carbon sources for polyhydroxyalkanoate (PHA) biosynthesis in *Cupriavidus necator* and related organisms, enabling the production of biodegradable bioplastics from citrus-waste-derived feedstocks.

Succinic acid fermentation by *Actinobacillus succinogenes* achieves productivities of 25–35 g·L^−1^ from citrus-derived glucose under CO_2_-enriched conditions, with yields of 0.7–0.8 g per gram of substrate [61]. Succinic acid serves as a building block for polybutylene succinate (PBS) and other bio-based polymers, positioning citrus biorefineries as suppliers to the growing bioplastics sector.

The biotransformation of D-limonene into higher-value terpenoids represents an emerging valorisation strategy, with *Penicillium digitatum* and related fungi converting limonene to α-terpineol with >95% regioselectivity [141]. This biocatalytic approach produces fragrance and pharmaceutical intermediates commanding substantially higher prices than the parent monoterpene.

Biological stabilisation of residual organic matter completes the biorefinery cycle, converting materials unsuitable for extraction or fermentation into soil amendments [149,151]. Aerobic composting of mixed citrus residues achieves 40–50% mass reduction over 8–12 weeks, producing mature compost with C:N ratios of 15–20 suitable for horticultural applications [149]. Vermicomposting with *Eisenia fetida* further enriches nutrient content whilst generating humic substances that enhance soil structure and fertility [151]. Anaerobic digestate, following dewatering and stabilisation, provides a mineralised biofertiliser containing plant-available nitrogen, phosphorus, and potassium, thereby returning nutrients to agricultural production and closing the circular-economy loop [61,146].

The integration of these biotechnological pathways within cascade-biorefinery configurations—wherein extraction residues become fermentation substrates, fermentation residues undergo composting, and process effluents generate biogas—maximises total value recovery whilst minimising waste generation, exemplifying circular-bioeconomy principles in industrial practice.

## 7. Green Extraction Technologies

**Figure 7:**
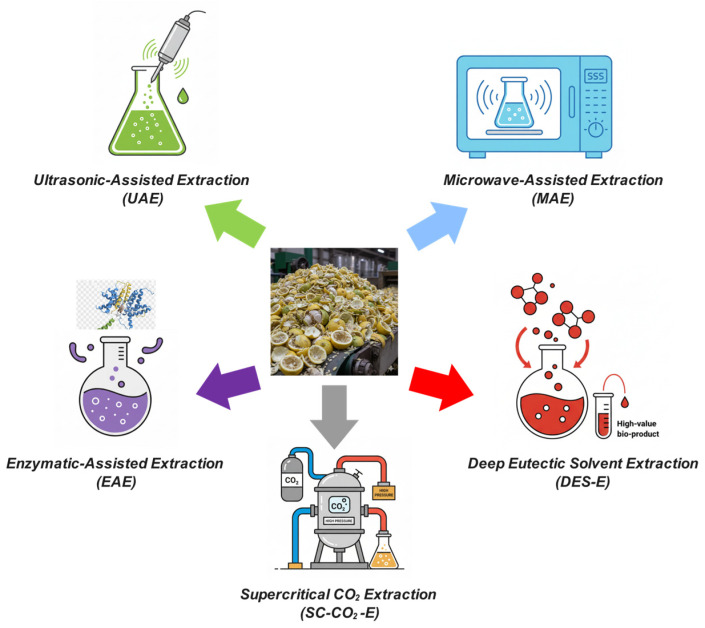
Green extraction technologies for valorisation of lemon-processing residues. Schematic overview of sustainable extraction methodologies—ultrasound-assisted (UAE), microwave-assisted (MAE), enzyme-assisted (EAE), deep eutectic solvent (DES-E), and supercritical CO_2_ (SC-CO_2_-E) extraction—employed in cascade-biorefinery configurations for recovery of high-value bioproducts from citrus waste streams.

The citrus-processing industry generates 15–32 million tonnes of waste annually, representing 50–60% of raw fruit mass [138,163,182,212], yet green extraction technologies can transform this environmental burden into high-value bioactive compounds, including essential oils, pectin, and polyphenols, with yields 30–112% higher than conventional methods. Recent advances (2020–2025) in ultrasound-assisted extraction, microwave-assisted extraction, supercritical fluid extraction, and enzyme-assisted extraction have demonstrated remarkable efficiency improvements: ultrasound achieves 89% time reduction [213] with 50% yield enhancement [214,215], microwave technology reduces energy consumption 27-fold [18], supercritical CO_2_ produces 95–99% pure limonene whilst remaining solvent-free [163,212], and enzymatic approaches selectively release bioactive aglycones with 95% conversion efficiency under mild conditions. These sustainable technologies operate synergistically—sequential extraction maximises compound recovery whilst minimising environmental impact—positioning the citrus industry at the forefront of implementing the circular bioeconomy (Figure 7).

The convergence of these green methodologies with natural deep eutectic solvents and process-intensification strategies promises to revolutionise citrus-waste valorisation, offering economically viable pathways to produce pharmaceutical-grade bioactives, food-grade pectin, and natural antioxidants whilst eliminating the toxic solvents and harsh conditions characteristic of conventional extraction.

### 7.1. Ultrasound-Assisted Extraction

Ultrasound-assisted extraction has emerged as one of the most promising green technologies for citrus-waste valorisation, operating via acoustic cavitation that generates localised conditions of approximately 5000 K and 1000 atmospheres [212,216]. When ultrasonic waves (typically 20–40 kHz) propagate through the extraction medium, they generate alternating compression and rarefaction cycles, which form microscopic bubbles. The violent collapse of these bubbles produces shock waves that fragment cell walls, induce sonoporation in cellular membranes, and dramatically enhance mass transfer through turbulence and shear forces [216]. This mechanical disruption, combined with increased water absorption and tissue swelling, enables solvent penetration deep into cellular structures without requiring elevated temperatures or harsh chemical conditions.

Recent studies have demonstrated that pretreatment strategies significantly influence both extraction efficiency and the preservation of thermolabile bioactive compounds. For instance, Martínez-Abad et al. [130] optimised a cascade-valorisation approach for lemon-peel residues using microwave-assisted processes, achieving sequential recovery of essential oils and flavonoid-rich pigments under controlled conditions that minimise degradation. Complementary evidence indicates that weak-acid pretreatment (pH 1.5–2.5, 60–80 °C) facilitates pectin gelation and partial cell-wall disruption without inducing Maillard reactions, thereby enhancing subsequent ultrasound-assisted extraction (UAE) performance. This combined approach yields food-grade pectin with a degree of esterification of 55–65 while preserving eriocitrin at recovery rates exceeding 90%, compared with markedly lower retention under conventional boiling extraction. Such mechanistic insights underscore the critical role of pretreatment in enabling selective extraction and maintaining bioactive integrity within integrated biorefinery frameworks.

Recent investigations by Panwar et al. [37] optimised the ultrasound-assisted extraction of pectin from *Citrus limetta*, achieving a maximum yield of 28.8% at 40 °C, 37% ultrasonic amplitude, 24 min, and pH 1.9 using citric acid; the degree of esterification obtained was approximately 55.3%. Comparative analysis revealed that UAE enhanced pectin yield by 16–31% relative to conventional extraction, whilst reducing processing time from several hours to 20–45 min [212,215].

For polyphenol recovery, Thiruvalluvan et al. [217] optimised UAE conditions for sweet-lime-peel waste using response surface methodology, identifying optimal parameters of 60% ethanol, 60 °C, 40 min, and 1:20 solid–liquid ratio that yielded 1522–1812 mg gallic acid equivalents (GAE) per 100 g for lemons and up to 15,256 mg GAE per 100 g for mandarin peels, representing a 38–50% yield improvement over conventional solvent extraction.

The extraction of essential oils, particularly D-limonene, is another primary application of UAE technology [218]. Ultrasound-assisted extraction (UAE) applied to citrus residues offers advantages such as enhanced efficiency, reduced processing time, and improved preservation of volatile compounds compared with conventional methods [212]. This method employs ultrasonic frequencies of approximately 20–25 kHz and operates at temperatures not exceeding 60 °C for essential oils [219]. The mechanism involves preferential disruption of oil glands in citrus peel, releasing terpenes and monoterpenes without thermal degradation.

Abdallah et al. [220] applied the Box–Behnken response surface methodology to optimise the extraction of polyphenols and flavonoids from *Citrus aurantium* var. amara peel, achieving optimal conditions of 50% ethanol, 60 °C, and 30 min that maximised the total phenolic content while preserving antioxidant capacity.

The study identified hesperidin as the predominant flavonoid (187.6–6444 mg per 100 g depending on citrus variety), followed by naringin (1.1–4203 mg/100 g), eriocitrin (20.71 mg·g^−1^), and diosmin (18.59 mg·g^−1^) [221]. These flavonoid glycosides possess significant pharmaceutical potential, including anti-inflammatory, antioxidant, and cardiovascular-protective properties. Notably, UAE preserved the structural integrity of these heat-sensitive compounds by operating at moderate temperatures, in contrast to conventional boiling extraction, which degrades thermolabile bioactives.

Recent innovations have focused on using natural deep eutectic solvents (NaDES) to replace conventional organic solvents entirely [222]. Dominguez-Rodriguez et al. [222] demonstrated that sequential extraction—using supercritical CO_2_ for terpenoids, followed by UAE with choline chloride–tartaric acid NaDES for polyphenols —provided comprehensive recovery of bioactives from grapefruit and lemon peels while maintaining green chemistry principles. The NaDES-UAE approach achieved yields comparable to those of ethanol-based systems, while significantly reducing environmental toxicity and improving biodegradability. This represents a paradigm shift towards truly sustainable extraction, eliminating reliance on petroleum-derived solvents. Additionally, pulsed UAE operation has demonstrated 50% energy savings compared with continuous sonication, whilst maintaining extraction efficiency through duty-cycle optimisation [223].

The techno-economic advantages of UAE extend beyond yield improvements. Energy consumption is reduced by 50–70% compared to conventional extraction, whilst solvent usage decreases by 30–50% due to enhanced mass-transfer efficiency [216]. Processing temperatures of 40–75 °C, rather than 80–100 °C for conventional methods, result in lower operating costs and better preservation of bioactive quality [212]. Anticona et al. [212] comprehensively reviewed UAE applications of citrus waste, emphasising that technology enables the implementation of a circular economy by transforming disposal costs into revenue streams through the recovery of pharmaceutical-grade hesperidin, food-grade pectin, and natural antioxidants for the cosmetics industry.

### 7.2. Microwave-Assisted Extraction

Microwave-assisted extraction operates through a fundamentally different mechanism from conventional heating, utilising 2.45 GHz electromagnetic radiation that interacts directly with polar molecules within the plant matrix [51,59,162,212]. The dielectric heating mechanism involves both ionic conduction and dipole rotation, in which microwaves induce rapid oscillations of water molecules and other polar compounds, converting electromagnetic energy directly into kinetic energy and heat. This volumetric heating generates localised pressure from the expansion of water vapour within intact cell walls, causing mechanical rupture and dramatically increased permeability, thereby facilitating the diffusion of bioactive compounds into the extraction solvent [168]. Unlike conductive heating in conventional extraction, where heat transfers slowly from the outside to the inside of the sample, microwave energy penetrates throughout the matrix simultaneously, enabling extraction times measured in minutes rather than hours.

Martinez-Abad et al. [130] optimised a sequential MAE process for lemon-peel waste, which first extracted essential oil via microwave-assisted hydrodistillation using a Clevenger apparatus, followed by pigment extraction from the residual material. The optimal water-to-solid ratio of 1:1.5, with a total extraction time of 20 min at a constant pressure of 300 mbar, yielded approximately 2.0 wt% essential oil, containing 65.08% D-limonene, 14.52% β-pinene, and 9.74% γ-terpinene. Gas chromatography−flame ionisation detection identified 65 compounds in the essential oil, which exhibited potent antimicrobial activity against *Escherichia coli* and *Staphylococcus aureus* [130]. Subsequent pigment extraction from the spent peel using 80% ethanol at 80 °C for 50 min yielded approximately 6 wt% pigment, demonstrating a cascade-valorisation approach that maximises resource utilisation from citrus waste [130].

For pectin extraction, MAE has demonstrated exceptional efficiency and product quality. Duggal et al. [168] conducted comprehensive optimisation of microwave-assisted acid extraction from kinnow (*Citrus reticulata*) peels using response surface methodology, identifying optimal conditions of 110 °C, pH 2.2 with 1% acetic acid, 10 min extraction time, and a pulse ratio of 1 (10 s on/10 s off) that yielded 9.81% pectin with a 66.67% degree of esterification classified as a high-methoxyl pectin suitable for food-gelling applications. The extracted pectin exhibited a galacturonic acid content of 63.15%, exceeding commercial citrus pectin (60.63%), alongside superior functional properties, including a water-holding capacity of 8.27 g·g^−1^ versus 6.16 g·g^−1^ for commercial pectin, oil-holding capacity of 3.10 g·g^−1^ versus 2.61 g·g^−1^, and water-swelling capacity of 20 mL·g^−1^ versus 8 mL·g^−1^ [168]. Fourier transform infrared spectroscopy confirmed the characteristic pectin peaks, while scanning electron microscopy revealed a compact, wrinkled, and porous structure that is optimal for hydration. Thermogravimetric analysis showed a maximum decomposition temperature range of 200–700 °C, accompanied by a 72.5% weight loss, confirming thermal stability suitable for food-processing applications [168].

The life-cycle assessment conducted by Duggal et al. [168] provided critical insights into the environmental sustainability of MAE. The analysis identified ethanol used for pectin precipitation as contributing 49% of the climate-change impact, followed by acetic acid extraction solvent. However, MAE demonstrated a substantially lower environmental footprint than conventional extraction across all impact categories, including climate change, freshwater eutrophication, human toxicity, ionising radiation, and ozone depletion [168]. The study recommended three strategies to reduce environmental impact further: (1) substituting bioethanol from cane or beet molasses with fossil-derived ethanol, achieving a 25–11% reduction in climate impact; (2) concentrating the extract before precipitation to reduce ethanol consumption by 80%; and (3) implementing ethanol recycling via distillation with a 76% recovery efficiency [168]. These modifications could transform MAE into a truly sustainable technology with minimal environmental burden.

Juric et al. [169] demonstrated an integrated biorefinery approach for mandarin peel using MAE to sequentially extract polyphenols/carotenoids followed by pectin from the residue. Response surface methodology optimisation revealed that the sample-to-solvent ratio and solvent type were the most significant factors for polyphenol/carotenoid extraction. In contrast, extraction time and microwave power were critical for pectin yield [169]. The optimal extract contained 21.76 ± 0.46 mg GAE per gram of total polyphenols, 139.7 ± 2.28 mg per gram of tangeretin, and 703.62 ± 51.72 μg per gram of nobiletin, accompanied by substantial carotenoid recovery. The extracts exhibited high DPPH and ABTS radical-scavenging capacities, confirming the preservation of antioxidant functionality [169]. A comparative life-cycle assessment demonstrated that MAE had more than a two-fold-lower environmental impact than conventional solvent extraction across all sustainability indicators, supporting the implementation of a circular economy through a citrus-waste biorefinery [169].

Energy efficiency represents perhaps the most compelling advantage of MAE technology. Comparative studies have documented that MAE requires only 122–180 s processing time versus 7200 s for conventional solvent extraction, representing a 95–98% time reduction [212]. Solventless MAE for essential-oil extraction has demonstrated substantially lower energy consumption than conventional hydrodistillation, with energy requirements reduced by 80–91%, while achieving 95.2% limonene purity in essential oils extracted from orange peel [212]. Furthermore, the development of completely solvent-free microwave-extraction techniques eliminates the need for organic solvents, representing a paradigm shift towards zero-waste processing [162]. These performance improvements, combined with significant reductions in processing time and the elimination of hazardous solvent consumption, position MAE as an economically attractive technology for industrial implementation in citrus-waste valorisation.

However, MAE technology presents specific limitations that require careful process control. Extended microwave exposure beyond optimal conditions leads to thermal degradation of pectin chains and bioactive compounds. Studies have documented that extraction at power levels exceeding 600 W or lasting more than 10 min for pectin significantly reduces yields due to chain scission and depolymerisation [215]. For lemon- and mandarin-peel extraction, microwave treatment exceeding 1 min at 360 W produced viscous mixtures that could not be centrifuged, whereas prolonged processing degraded the pectin molecular structure [215]. Consequently, pulse-mode operation (typically 10 s on/10 s off) represents the best practice for minimising spillage, controlling temperature, and preventing compound degradation while maintaining extraction efficiency.

### 7.3. Supercritical Fluid Extraction

Supercritical fluid extraction using carbon dioxide represents the pinnacle of selective, environmentally benign extraction technology for valorising citrus waste. Supercritical CO_2_ exists above its critical point (31.1 °C, 7.38 MPa), exhibiting unique physicochemical properties that combine gas-like diffusivity with liquid-like density, while maintaining zero surface tension, which enables deep penetration into solid matrices. The density of SC-CO_2_ can be precisely modulated by adjusting pressure and temperature, providing exquisite control over solvent power and selectivity. This tunability enables the sequential extraction of compounds with different polarities—first extracting non-polar terpenes with pure SC-CO_2_ at lower pressures, then increasing pressure and adding an ethanol co-solvent to recover moderately polar flavonoids, and finally employing high pressure with higher ethanol concentrations for polar phenolic acids [164].

Mai et al. [163] developed a sophisticated two-stage SC-CO_2_ extraction process for *Citrus grandis* peel, which first recovered essential oil using pure SC-CO_2_ at 100 bar and 40 °C for 300 min, followed by naringin extraction at 120 bar and 50 °C for 2 h with an 80% ethanol co-solvent. The essential oil contained 95.66% D-limonene, representing a 13-fold-higher purity than conventional hydrodistillation (87.60%), accompanied by β-pinene (1.51%), α-phellandrene (1.13%), and α-pinene (0.90%) [163]. The naringin yield was 3.8% under optimal conditions, corresponding to pharmaceutical-grade purity. Antimicrobial assessment demonstrated minimum inhibitory concentrations of 0.25 mg·mL^−1^ against *Moraxella catarrhalis* and 1.0 mg·mL^−1^ against *Streptococcus pyogenes* and *Streptococcus pneumoniae* [163], whilst antifungal activity against *Trichophyton rubrum*, *T. mentagrophytes*, and *Microsporum gypseum* exhibited MIC values of 6.25–12.5 μM for naringin [163]. The separation of extraction stages prevented cross-contamination between volatile terpenes and non-volatile flavonoids, allowing for the targeted recovery of distinct compound classes.

Romano et al. [164] systematically compared liquid CO_2_ and supercritical CO_2_ extraction from orange, tangerine, and lemon peels, revealing critical insights into co-solvent requirements. Pure CO_2_, whether liquid (20 MPa, 20 °C) or supercritical (30 MPa, 60 °C), yielded less than 1.5% due to the apolar nature of CO_2_ being insufficient to extract polar compounds effectively. However, the addition of 20% ethanol co-solvent dramatically enhanced extraction, with liquid CO_2_ + 20% ethanol achieving the highest yields: orange peel 35.16%, tangerine 28.59%, and lemon 30.69% relative to 100% ethanol controls [164]. For lemon peel specifically, liquid CO_2_ extraction with 20% ethanol yielded 43.84% limonene and 19.86 mg/g dry-matter naringin, alongside a substantial sesquiterpene content, including bergamotene (7.27–15.10%), bisabolene (8.23–21.06%), and caryophyllene (3.55–6.70%). Total flavonoid content reached 5816.9 μg·g^−1^ dry matter. The study demonstrated that extraction conditions profoundly influence compound profiles, with liquid CO_2_ conditions proving more efficient than supercritical CO_2_ for certain flavonoids, despite theoretical predictions [164].

The environmental and sustainability advantages of SC-CO_2_ extraction have positioned it as the gold standard for green chemistry applications [224]. Argun et al. [224] pioneered the application of SC-CO_2_ to citrus-processing wastewater rather than solid waste, demonstrating that countercurrent SC-CO_2_ extraction at optimal conditions of 28.7 MPa and 60 °C could recover water-soluble bioactive compounds while simultaneously reducing wastewater toxicity. Phenolic-compound concentrations increased between 2- and 260-fold in the extracts, including hesperetin, quercetin, apigenin, cyanidin, p-coumaric acid, ferulic acid, sinapic acid, and isorhamnetin derivatives [224]. Total phenolic content maximised at optimal pressure and temperature, whilst total flavonoid content exhibited inverse proportionality to pressure. Critically, wastewater toxicity classification improved from “very toxic” (Class IV) to “toxic” (Class III) following extraction, demonstrating that SC-CO_2_ technology provides the dual benefits of bioactive-compound recovery and pollution reduction [224]. This pioneering work established SC-CO_2_ as a viable option for liquid waste streams, expanding its applications beyond solid citrus-peel processing [224].

Rajput et al. [225] optimised SC-CO_2_ extraction of essential oils from kinnow (*Citrus reticulata*) peels using Box–Behnken response surface methodology across temperature ranges of 35–55 °C, pressure ranges of 200–350 bar, and extraction times of 60–150 min. The optimal conditions, 43 °C, 297 bar, and 120 min, yielded 1.57% essential oil with 79.94% DPPH radical-scavenging activity and a total phenolic content of 41.22 mg GAE per gram [225]. The mathematical model demonstrated excellent predictive capability, with high *R*^2^ values, enabling process optimisation without the need for extensive experimental trials. The extracted essential oils contained antioxidants and polyphenols with demonstrated antimicrobial activity, making them suitable for applications in food preservation, pharmaceutical formulations, and cosmetic products [225]. The study emphasised that kinnow peels, rich in oil glands, represent an underutilised resource that SC-CO_2_ extraction can transform into high-value products [225].

Sequential and integrated extraction strategies represent the cutting edge of SC-CO_2_ technology development [222]. Dominguez-Rodriguez et al. [222] developed sustainable strategies that combine SC-CO_2_, ultrasound-assisted extraction, and natural deep eutectic solvents for grapefruit, lime, and lemon peels [222]. The sequential approach employed SC-CO_2_ first to extract the terpenoid-rich essential-oil fraction, followed by UAE with NaDES (choline chloride-based system) to recover phenolic compounds from the defatted residue [222]. This strategy provided holistic exploitation of citrus peels by separating non-polar and polar compound classes, maximising total bioactive recovery whilst minimising solvent consumption. SC-CO_2_ extracts exhibited rich terpenoid profiles with vigorous anticholinergic activity, whilst UAE-NaDES extracts contained high concentrations of naringin from grapefruit and hesperidin from lime and lemon, alongside diverse phenolic compounds [222]. The combined approach achieved greater phenolic diversity than direct UAE-NaDES extraction, demonstrating that SC-CO_2_ pretreatment enhanced subsequent extraction efficiency by disrupting the matrix and reducing particle size.

The principal limitation of SC-CO_2_ technology remains capital investment and operational costs [226,227]. High-pressure vessels, specialised pumps, and safety systems require substantial initial expenditure, whilst pressurisation energy costs represent ongoing operational expenses [228]. Extraction times of 2–5 h for complete recovery, whilst shorter than those required by some conventional methods, exceed the minutes required for microwave or ultrasound extraction [228]. Additionally, pure CO_2_ is ineffective for highly polar compounds, necessitating the addition of a co-solvent, which introduces additional separation requirements [229]. However, these limitations must be weighed against the advantages of SC-CO_2_: solvent-free final products that require no post-extraction purification, preservation of thermolabile compounds at low operating temperatures, a recyclable solvent with GRAS status for food applications, and unmatched selectivity for targeted compound recovery [228]. For high-value applications that demand maximum purity—such as pharmaceutical formulations, premium essential oils, and functional-food ingredients—SC-CO_2_ extraction justifies the higher costs through superior product quality and the complete absence of solvent residues [228,230].

### 7.4. Enzyme-Assisted Extraction

Before examining enzyme-assisted extraction methodologies, it is essential to clarify the conceptual distinction between this approach and the biotechnological production of industrial enzymes discussed in Section 5.2. Enzyme-assisted extraction (EAE) employs hydrolytic enzymes, whether commercially sourced or produced in-house, as processing aids to enhance the recovery of bioactive compounds already present in the plant matrix. The enzymes function as catalytic tools that selectively degrade cell-wall polysaccharides (pectin, cellulose, hemicellulose), thereby increasing tissue permeability and facilitating the release of intracellular phenolics, flavonoids, and other target metabolites. In this context, EAE operates as a green extraction technology alongside UAE, MAE, and SC-CO_2_, sharing the common objective of maximising bioactive recovery whilst minimising environmental impact. The enzymes themselves are consumed during processing rather than constituting the final product. This contrasts fundamentally with the enzyme production pathway (Section 5.2), in which lemon residues serve as fermentation substrates and enzymes constitute the primary value-added output. Recognising this distinction enables rational integration of both strategies within cascade-biorefinery configurations: enzymes produced from one waste fraction may subsequently be employed to enhance extraction efficiency from other fractions, thereby creating synergistic process linkages.

Enzyme-assisted extraction represents a paradigm shift from physical and chemical disruption methods to biological catalysis, employing specific hydrolytic enzymes that target the complex carbohydrate matrix of citrus-peel cell walls. The fundamental mechanism involves enzymatic hydrolysis of glycosidic bonds in pectin, cellulose, hemicellulose, and arabinoxylan, thereby transforming the rigid plant cell wall into a porous structure with enhanced solvent accessibility [231,232]. Beyond mere structural disruption, EAE provides selective biotransformation of glycosylated flavonoids into their aglycone forms, which exhibit superior bioavailability, enhanced biological activity, and higher commercial value for pharmaceutical and nutraceutical applications [233]. This dual functionality—simultaneous extraction and bioconversion—distinguishes EAE from purely mechanical extraction technologies.

In the revised manuscript, enzymatic pretreatment is described as the controlled hydrolysis of galacturonic acid linkages in pectin and β-1,4-glucosidic bonds in cellulose by pectinase–cellulase cocktails, which converts the rigid cell wall into a porous, highly permeable matrix that facilitates ultrasound-assisted recovery of eriocitrin at mild, sub-degradative temperatures for flavonoid glycosides [216]. In parallel, a comparative analysis of cold pressing versus chemical pretreatment for lemon seeds is presented, emphasising that cold-pressed oils typically yield slightly less oil but retain a higher proportion of native tocopherols and phenolic antioxidants than solvent-based or more intensive mechanical extraction methods [234].

The enzyme types critical for citrus-waste processing span multiple hydrolase classes that operate synergistically. β-Glucosidases (EC 3.2.1.21) represent the most valuable enzymes, hydrolysing β-1,4-glycosidic bonds in both cellobiose and flavonoid glycosides, thereby releasing phenolic aglycones from their sugar moieties. Cellulases, including endo-β-1,4-glucanases (EC 3.2.1.4), cleave internal bonds in cellulose chains, whilst cellobiohydrolases (EC 3.2.1.91) processively release cellobiose units from crystalline cellulose. Pectinases—polygalacturonases, pectin lyases (EC 4.2.2.10), and pectin methylesterases (EC 3.1.1.11)—target the pectin fraction that constitutes 20–30% of citrus-peel dry weight [232]. Hemicellulases, particularly endo-β-1,4-xylanases (EC 3.2.1.8) and β-xylosidases (EC 3.2.1.37), degrade xylan and other hemicelluloses, whilst amylases hydrolyse residual starch components [235].

Díaz et al. [236] achieved a breakthrough by developing a low-cost enzymatic cocktail from *Aspergillus niger* LBM 134, cultivated on sugarcane bagasse—a common agricultural-waste substrate. The homemade enzyme preparation exhibited β-glucosidase activity of 11.97 ± 0.10 U·mL^−1^, with optimal performance at 50 °C and pH 5, retaining 70% residual activity after 72 h at 50 °C [236]. The optimised extraction conditions of 40 °C, pH 5.0, 8 h, 10 IU β-glucosidase per gram of biomass, and 2% substrate concentration yielded 71.97 ± 1.71 mg GAE per mL of total phenolic content, representing a 112% higher yield than alkaline extraction and a 30% higher yield than the commercial *Viscozyme* L enzyme [236]. High-performance liquid chromatography analysis identified hesperetin, quinic acid, p-coumaric acid, gallic acid, and tryptophan as the major compounds extracted. Critically, the production cost of this fungal enzymatic cocktail was 150 times lower than that of commercial enzymes, dramatically improving the economic viability of industrial-scale EAE implementation for citrus-waste valorisation [236].

Barbosa et al. [233] conducted a rigorous investigation into the effects of β-glucosidase, tannase, and cellulase on citrus by-products, demonstrating that β-glucosidase at 20 U per gram of substrate for 24 h achieved optimal bioconversion. The enzymatic treatment dramatically altered polyphenolic profiles, converting hesperidin to hesperetin (766.44 mg per 100 g) and narirutin to naringenin (77.63 mg per 100 g), with 95% conversion efficiency for hesperidin biotransformation [233]. These aglycone forms exhibit substantially higher biological activity than their parent glycosides due to improved cellular absorption and bioavailability [233]. The enzymatically treated extracts demonstrated enhanced antibacterial activity against pathogenic bacteria and improved prebiotic properties in Caco-2 cell-culture models, stimulating beneficial bacterial growth whilst inhibiting pathogens and modulating cytokine production [233]. This research established that EAE not only extracts compounds but fundamentally transforms their biological properties through selective enzymatic modification.

The identification of superior microbial strains for enzyme production represents a key area of active research. Gooruee et al. [235] screened multiple *Trichoderma* species for extracellular enzyme production capability on lemon-peel waste, identifying *Trichoderma afroharzianum* NAS107 as the superior strain. Cultivated at 28 °C and pH 5 with optimal enzymatic activity observed at 96–120 h, this strain produced a comprehensive enzyme profile including xylanase, multiple exoglucanases (Cel 6A, Cel 7A), endoglucanases (Cel 5A, Cel 12A), β-glucosidases (Cel 3A, Cel 1A), polygalacturonases I and II, pectin lyase, and pectin esterases I and II [235]. The synergistic action of these enzymes proved particularly effective for saccharifying citrus industrial waste, demonstrating that microbial-enzyme cocktails provide naturally balanced ratios of complementary activities, optimised through evolutionary adaptation to lignocellulosic substrates.

Process intensification through the combination of EAE with other green technologies has demonstrated substantial synergistic benefits. Ultrasound-assisted enzymatic extraction (UAEE) utilises 20–40 kHz ultrasound to generate cavitation, which disrupts cell structures, thereby enhancing enzyme accessibility to intracellular substrates and improving the mass transfer of released compounds [237,238]. Sequential application—ultrasound pretreatment for 15–30 min followed by enzymatic hydrolysis for 4–8 h—reduces total processing time by 50–70% compared to EAE alone, whilst improving yields by 20–40% [123].

Economic and environmental considerations ultimately determine the potential for industrial adoption. While enzyme costs of USD 500–2000 per kilogram for commercial preparations represent a significant expense at typical usage levels of 10–40 U per gram of substrate, the development of low-cost microbial-enzyme production using agricultural-waste substrates dramatically alters the cost–benefit calculations [239]. The demonstration by Díaz et al. [236] of a 150-fold cost reduction through *Aspergillus niger* enzyme production from sugarcane bagasse, combined with yields 30% higher than those of commercial enzymes, suggests that in-house enzyme production at processing facilities could achieve economic viability.

### 7.5. Comparative Assessment Reveals Complementary Strengths Across Green Technologies

The four green extraction technologies examined—ultrasound, microwave, supercritical fluids, and enzymes—display distinct mechanistic approaches and complementary performance characteristics, enabling strategic selection based on target compounds, required purity, processing scale, and economic constraints [240,241,242].

Ultrasound-assisted extraction enables rapid processing (20–60 min) with moderate capital investment, yielding 16–50% improvements over conventional methods through acoustic cavitation and enhanced mass transfer [212,216]. Technology excels for polyphenol extraction, preserving antioxidant activity, and offers excellent scalability and straightforward integration into existing processing lines. However, UAE provides limited selectivity, extracting broad compound classes simultaneously rather than targeted fractions.

Microwave-assisted extraction is a speed champion, completing extractions in 1–20 min with unprecedented energy efficiency—a 27-fold reduction compared to conventional hydrodistillation—through volumetric dielectric heating [18,212]. MAE is particularly suited to pectin extraction, producing high-quality products with superior functional properties (water-holding capacity of 134% of commercial pectin) whilst reducing processing time by 95% [168]. Life-cycle assessment confirms a substantially lower environmental impact across all sustainability indicators [168]. Yet, MAE risks thermal degradation of heat-sensitive compounds at excessive power levels, necessitating careful parameter optimisation and pulse-mode operation to prevent quality deterioration.

Supercritical CO_2_ extraction achieves unmatched selectivity and purity, producing 95–99% pure limonene and pharmaceutical-grade flavonoids through pressure and temperature modulation, while maintaining a complete absence of solvent residues in the final products [163]. The technology’s operation at low temperatures (40–60 °C) preserves thermolabile bioactives, whilst CO_2_’s GRAS status enables direct food and pharmaceutical applications without additional purification. Sequential extraction protocols systematically recover distinct compound classes from a single feedstock, maximising resource utilisation. However, high capital costs (USD 500,000–2,000,000 for industrial-scale equipment) and longer extraction times (2–5 h) constrain economic viability to high-value applications where superior purity justifies premium pricing.

Enzyme-assisted extraction offers a unique biotransformation capability, selectively converting flavonoid glycosides to their aglycone forms with 95% conversion efficiency under mild conditions (40–50 °C, pH 4.5–5.5), thereby preserving bioactive functionality. The β-glucosidase-catalysed transformation of hesperidin to hesperetin yields products with substantially enhanced bioavailability and biological activity compared to parent glycosides [110,233,243]. Recent advances in low-cost enzyme production from agricultural waste (a 150-fold cost reduction) have transformed economic feasibility [236], whilst synergistic combinations with ultrasound or microwave technologies accelerate processing and improve yields. Nevertheless, longer processing times (8–24 h) and the requirement for enzyme specificity present obstacles to broad industrial implementation.

A quantitative comparison reveals performance trade-offs across multiple dimensions. Processing times range from 1 to 20 min for MAE, 20 to 60 min for UAE, 2 to 5 h for SFE, and 8 to 24 h for EAE. Energy consumption measurements indicate a 27-fold reduction for MAE relative to conventional methods, and a 50–70% reduction for UAE [18]. Whilst SFE requires substantial energy for pressurisation, it is offset by the elimination of downstream-solvent recovery. Extraction yields demonstrate 9–36% for pectin (varying by citrus species and method) [30], 32.9 mg·g^−1^ limonene at 95–99% purity for SFE and UAE, and 21–72 mg GAE per gram of total phenolics, with EAE achieving the highest absolute values [168,215]. Capital investment ranges from USD 10,000–50,000 for laboratory UAE systems to USD 50,000–200,000 for MAE and EAE pilot plants, and up to USD 500,000–2,000,000 for industrial SFE installations.

Sequential and combined extraction strategies represent the frontier of innovation, systematically exploiting complementary technology strengths to maximise total bioactive recovery from citrus waste. The optimal cascade begins with SC-CO_2_ extraction of non-polar essential oils and terpenes [222], at 100–200 bar and 40–50 °C, followed by ultrasound-assisted extraction with natural deep eutectic solvents for polar phenolics, and concludes with enzymatic treatment or microwave-assisted extraction for pectin from the final residue. Dominguez-Rodriguez et al. [222] demonstrated that this integrated approach achieved higher total bioactive yields than any single technology, whilst minimising environmental impact through solvent recycling and waste reduction. The SC-CO_2_ pretreatment disrupted cellular architecture, enhancing subsequent UAE efficiency by 30–40%. In contrast, the sequential methodology produced distinct product streams suitable for various commercial applications, including pharmaceutical-grade essential oils, nutraceutical phenolic extracts, and food-grade pectin.

The selection of appropriate extraction technology is crucial for the efficient valorisation of lemon-processing residues within a biorefinery framework. Each technique presents distinct advantages and limitations with respect to extraction efficiency, energy requirements, environmental impact, and economic feasibility.

Table 9 provides a comprehensive comparative analysis of conventional extraction methods versus four emerging green technologies: ultrasound-assisted extraction (UAE), microwave-assisted extraction (MAE), supercritical CO_2_ extraction (SC-CO_2_), and enzyme-assisted extraction (EAE). This multi-dimensional assessment encompasses operational parameters, product-quality indicators, scalability potential, and techno-economic considerations to guide technology selection for industrial implementation (Table 9).

As evidenced in Table 9, green extraction technologies demonstrate substantial improvements over conventional methods across multiple performance dimensions. SC-CO_2_ extraction preserves bioactive compounds (95–99%) and poses minimal risk of thermal degradation, albeit with considerably higher capital investment requirements (300–4000 × 10^3^ USD). Conversely, MAE offers an attractive balance between extraction efficiency (75–90% yield), reduced processing time (1–10 min), and moderate capital costs (60–700 × 10^3^ USD), while achieving the highest GWP reduction (80–92%) relative to conventional processing. UAE and EAE represent intermediate alternatives, with EAE being particularly advantageous for obtaining high-purity pectin fractions under mild conditions. The target compound should ultimately guide the choice of technology, desired product quality, available infrastructure, and economic constraints specific to each biorefinery scenario.

### 7.6. Future Perspectives and Industrial-Implementation and Process-Design Scenarios

The convergence of green extraction technologies with Industry 4.0 concepts—real-time process monitoring, artificial intelligence optimisation, and digital twin modelling—has the potential to revolutionise the industrial-scale valorisation of citrus waste. Machine learning algorithms can optimise multiparameter extraction protocols more efficiently than traditional response surface methodology, identifying non-linear interactions and unexpected synergies across temperature, pressure, pH, enzyme concentration, ultrasound power, and solvent composition. Artificial neural network models developed for UAE optimisation have demonstrated superior predictive accuracy compared to conventional statistical approaches, enabling adaptive process control that responds to natural variation in feedstock composition across seasons and cultivars.

Process intensification through novel reactor designs represents another critical development pathway. Continuous-flow ultrasonic reactors with multiple transducer arrays achieve uniform cavitation intensity throughout large volumes, eliminating the dead zones characteristic of batch systems. Microwave reactors with advanced temperature and pressure monitoring prevent hotspots and thermal degradation whilst maximising energy-transfer efficiency. Supercritical fluid extraction systems incorporating in-line fractionation and selective precipitation enable real-time product separation, reducing downstream processing requirements.

The integration of green extraction technologies into comprehensive biorefinery concepts will prove essential for economic viability. The optimal industrial implementation processes citrus-peel waste through sequential stages: (1) essential-oil recovery via solventless microwave-assisted hydrodistillation or SC-CO_2_, yielding 1.5–2.5% high-purity limonene for flavouring and fragrance markets; (2) phenolic-compound extraction using ultrasound with recycled ethanol or natural deep eutectic solvents, producing pharmaceutical-grade hesperidin and naringenin for nutraceutical applications; (3) enzymatic or microwave-assisted pectin extraction with acid recovery and reuse, generating 10–30% food-grade pectin for gelling applications; (4) dietary fibre production from final residue through mechanical processing. This cascade maximises revenue through multiple product streams, transforming disposal costs estimated at USD 50–100 per tonne into potential revenues exceeding USD 500–1500 per tonne, depending on bioactive-compound market prices.

Techno-economic modelling for a hypothetical 10,000-tonne-per-year citrus-processing facility indicates that integrated green extraction implementation requires a capital investment of USD 8–15 million but generates projected annual revenues of USD 8–15 million (around USD 800–1500 per tonne) from recovered bioactives, achieving payback periods of 2–3 years. Operating costs—enzymes, solvents, energy, and labour—total approximately USD 3–6 million annually, depending on the selected technology mix, while avoided disposal costs contribute an additional USD 0.5–1.0 million in annual savings. Sensitivity analysis indicates that the market prices of hesperidin and naringenin exert a dominant influence on profitability, suggesting that pharmaceutical-grade product certification justifies additional quality-control investments, as these products command price premiums of 5–10 times those of food-grade extracts.

A structured implementation pathway can be articulated across three distinct scales.

• **Scenario A**—small-scale distributed processing (50–100 tonnes per annum)—prioritises modular UAE/MAE units with optional laboratory SC-CO_2_ capability, targeting pharmaceutical-grade essential oils and nutraceutical polyphenols; typical capital ranges from USD 250,000 to 450,000 with operating costs of USD 35–45 per tonne, revenues of USD 800–1200 per tonne, and payback in 3–4 years under sequential batch operation and manual preparation. Mass-balance calculations indicate that from 100 kg of lemon waste, approximately 2.5 kg of essential oil (with a 25% yield premium via MAE), 8 kg of polyphenols, and 18 kg of residual biomass are produced.
• **Scenario B**—medium-scale semi-continuous operation (500–1000 tonnes per annum)—integrates SC-CO_2_, UAE, and MAE with automated handling to deliver a balanced product portfolio (essential oils, standardised bioactive extracts, food-grade pectin). Indicative economics comprise USD 1.5–2.5 million in capital, USD 20–30 per tonne in operating costs, and USD 500–800 per tonne in revenues, achieving payback within 2–3 years. A representative mass balance for 1000 kg of lemon waste yields ~22 kg of essential oil (2.2 wt%), 72 kg of polyphenols, 150 kg of pectin, and 200 kg of post-extraction residue in a continuous-feed configuration with sequential batch modules.
• **Scenario C**—large-scale integrated industrial biorefinery (5000–10,000 tonnes per annum)—employs fully automated SC-CO_2_, continuous-flow UAE/MAE, solid-state fermentation for enzyme production, and anaerobic digestion, thereby enabling complete cascade valorisation. Capital requirements of USD 8–15 million and operating costs of USD 12–18 per tonne are offset by revenues of USD 800–1500 per tonne and payback periods of 2–3 years under continuous operation with real-time control. A 10,000 kg mass balance illustrates the recovery of ~220 kg of essential oil (2.2 wt%), 750 kg of polyphenols (7.5 wt%), and 1500 kg of pectin (15 wt%), along with enzymes, nanocellulose, and residues directed to biogas, highlighting economies of scale and process-integration benefits.

These differentiated investment profiles and techno-economic indicators underscore that biorefinery viability is conditioned by reliable bioavailability of citrus residues and by-products at volumes sufficient to mitigate supply risk and smooth utilisation across unit operations. In practice, the business case strengthens when feedstock assurance enables stable throughput aligned with each scenario’s configuration (batch for small-scale; semi-continuous for medium-scale; fully integrated continuous for large-scale), ensuring that expected yields, energy performance, and product portfolios are realised within the stated cost and payback envelopes.

**Figure 8:**
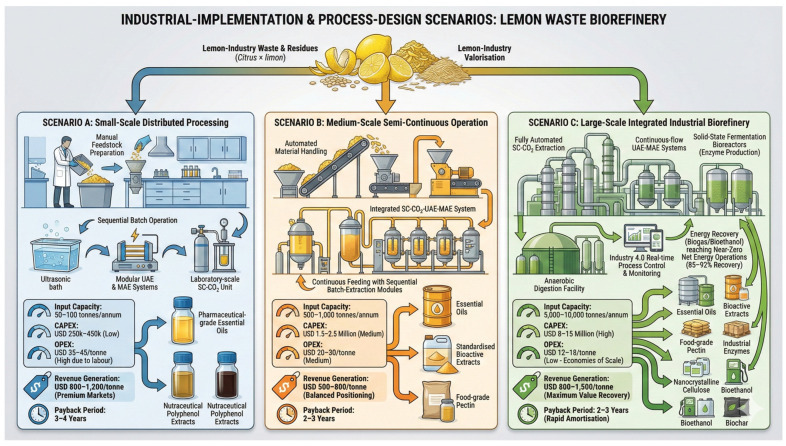
Lemon-waste biorefinery: industrial implementations and process-design scenarios.

Consequently, a prudent design principle is to engineer feedstock flexibility beyond a single citrus crop (e.g., lemon) by accommodating oranges, mandarins, and grapefruit within the same cascade framework. Such flexibility leverages the shared unit operations (SC-CO_2_, UAE, MAE, enzymatic steps) and the modularity noted in Scenario A, while preserving the comparative performance and portfolio logic in Scenario B and the integration advantages in Scenario C. By broadening eligible inputs across citrus residues, the facility reduces seasonal and regional supply volatility, optimises equipment utilisation, and enhances overall risk-adjusted returns across the three scenarios (Figure 8).

The environmental imperative for citrus-waste valorisation extends beyond greenhouse gas reduction and landfill diversion to encompass preventing water pollution and protecting biodiversity. Unprocessed citrus waste contains high concentrations of D-limonene and other monoterpenes that exhibit phytotoxicity and antimicrobial activity, rendering the waste unsuitable for direct agricultural application while causing aquatic toxicity when the leachate enters waterways. Green extraction technologies simultaneously recover these valuable compounds for commercial applications whilst detoxifying the residual material, enabling safe composting or animal-feed applications. Life-cycle assessments indicate that integrated biorefinery operations reduce overall environmental impact by 60–80% compared to conventional waste disposal, considering all factors, including energy consumption, chemical inputs, transportation, and the avoidance of synthetic-chemical production.

The emerging regulatory landscape increasingly favours naturally derived bioactive compounds over synthetic alternatives, driven by consumer preferences for “clean label” products and mounting evidence of biological activities absent in synthetic analogues. The European Union’s Farm to Fork strategy and circular economic action plan explicitly prioritise the valorisation of food waste and the development of a bio-based economy. At the same time, the United Nations Sustainable Development Goals emphasise responsible production and consumption patterns. These policy frameworks create favourable conditions for the adoption of green extraction technology, supported by research funding, tax incentives, and preferential procurement policies that reward sustainable practices. Consequently, the citrus-processing industry faces a strategic inflexion point at which environmental compliance, consumer demand, and economic opportunity converge, mandating a transition from disposal-focused to valorisation-focused waste-management paradigms.

### 7.7. Roadmap of Short-, Medium-, and Long-Term Research Priorities

**Figure 9:**
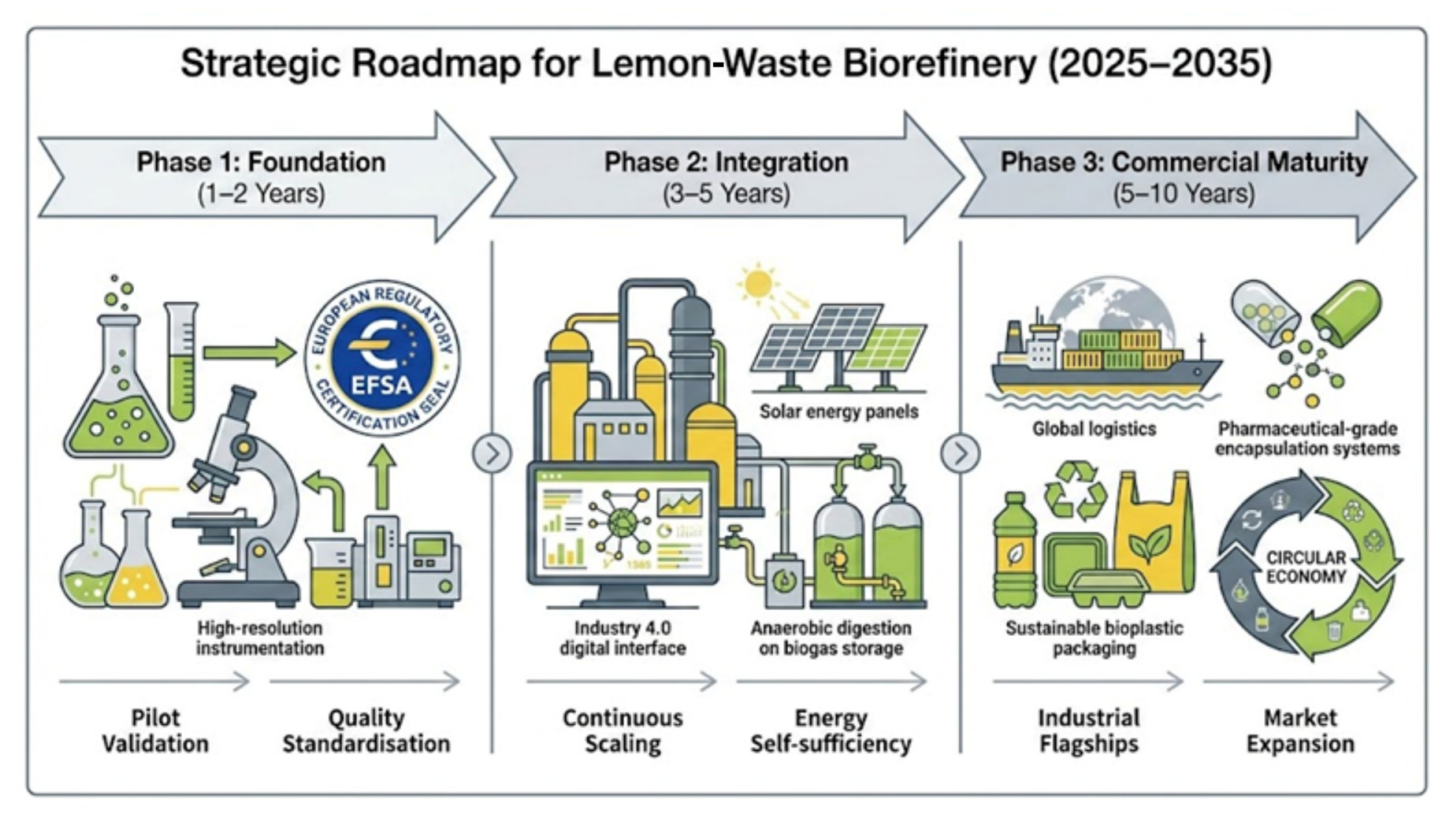
Strategic roadmap for lemon-waste biorefinery development (2025–2035). Phase 1 (1–2 years): pilot validation and quality standardisation; Phase 2 (3–5 years): continuous scaling and energy self-sufficiency; Phase 3 (5–10 years): industrial flagships and global market expansion.

The successful translation of laboratory-scale advances in lemon-waste valorisation into commercially viable, environmentally sustainable industrial biorefineries necessitates coordinated research, development, and deployment activities across multiple temporal horizons. This section delineates a structured roadmap encompassing immediate technical priorities (1–2 years), intermediate scale-up and market-development objectives (3–5 years), and long-term systemic-integration targets (5–10 years), as illustrated in Figure 9. The proposed roadmap addresses the critical research gaps identified in Section Identified Research Gaps whilst acknowledging the interdependencies between technological maturation, regulatory approval, market development, and infrastructure investment that collectively determine commercialisation success [69,70].

#### 7.7.1. Phase 1: Foundation (1–2 Years)

The immediate research agenda must prioritise experimental validation of integrated cascade-valorisation concepts through pilot-scale demonstrations processing 100–500 kg per batch [75,76]. These demonstrations should systematically evaluate sequential extraction protocols that combine supercritical CO_2_ for essential-oil recovery, followed by ultrasound- or microwave-assisted extraction for polyphenol fractions, and conclude with enzymatic or acid-assisted pectin isolation. Critical performance metrics—cumulative value recovery (USD per tonne feedstock), energy efficiency (kWh per kg product), and solvent-recycling efficiency—must be rigorously quantified under industrially relevant conditions.

Concurrently, standardised quality-assessment methodologies for emerging bioactive extracts require development and validation across multiple laboratories. Pharmaceutical-grade specifications should be established for the standardisation of hesperidin, eriocitrin, and naringin, including purity thresholds (≥95% for pharmaceutical applications), acceptable impurity profiles, and stability parameters [130,164]. Regulatory engagement with the European Food Safety Authority (EFSA), the U.S. Food and Drug Administration (FDA), and analogous agencies is a critical short-term priority, enabling the identification of specific toxicological studies and manufacturing quality-control requirements [69].

The establishment of the first commercial-scale demonstration facilities processing 500–2000 tonnes per annum in principal lemon-producing regions—Spain, Italy, Argentina, and Mexico—constitutes the capstone short-term priority [71,72]. These facilities must operate as living laboratories, documenting operational challenges, actual capital and operating costs, and the actualisation of profitability relative to techno-economic model predictions.

#### 7.7.2. Phase 2: Integration (3–5 Years)

The medium-term horizon emphasises the transition from batch-scale demonstrations to continuous, commercial-scale operations with Industry 4.0 integration [18,168]. Extraction technologies must be scaled to achieve processing throughput exceeding 500 kg per hour whilst maintaining validated product-quality specifications. Process-automation systems incorporating real-time, in-line analytical monitoring (e.g., near-infrared spectroscopy, fluorescence), adaptive control algorithms, and predictive maintenance scheduling should be developed to ensure consistent product quality despite natural variations in feedstock composition across seasons and cultivars.

Energy self-sufficiency represents a central medium-term objective. Solar thermal systems should be evaluated for low-temperature heating requirements (40–80 °C) prevalent in enzymatic and ultrasound-assisted extraction operations [212,216]. Biogas derived from the anaerobic digestion of final residual biomass can provide process heat and electricity through combined heat and power systems, enabling near-zero–net-energy operations [61,126]. The demonstration of energy-self-sufficient biorefineries positions citrus processing as an exemplar of sustainable industrial biotechnology.

Market development for emerging biorefinery products requires coordinated commercial and technical efforts. Pharmaceutical-grade polyphenol extracts, nanocrystalline cellulose composites, and speciality enzyme preparations must transition from technical samples to qualified commercial products, supported by established supply agreements [67,74]. Strategic partnerships with end-use manufacturers should be formalised to create stable market channels that justify biorefinery capital investments.

#### 7.7.3. Phase 3: Commercial Maturity (5–10 Years)

The long-term vision encompasses the establishment of 3–5 fully integrated commercial-scale biorefinery facilities processing 10,000+ tonnes per annum of lemon waste, distributed across major lemon-producing regions to minimise transportation costs and ensure a stable feedstock supply [71]. These flagship facilities must demonstrate economies of scale, rendering biorefinery products price-competitive with synthetic alternatives in mass-market applications, thereby enabling transition from niche premium markets to broad commercial deployment.

Global market expansion requires regulatory approval pathways for novel biorefinery ingredients and nanomaterials across major jurisdictions (European Union, United States, China, India, Brazil), with efforts directed toward regulatory harmonisation, facilitating international commercialisation [69]. The development of secondary biorefinery applications—nanocellulose-reinforced bioplastics for packaging, pharmaceutical-grade encapsulation systems, and bio-based polyols—expands market opportunities beyond the traditional food and pharmaceutical sectors [200,201,205].

Full circular-economy integration positions lemon biorefineries within broader industrial symbiosis networks with adjacent food-processing industries. Shared processing infrastructure, amortised across multiple feedstock sources, improves capital utilisation whilst providing operational resilience against seasonal variations [58,72]. The transformation of lemon-processing waste from disposal liability to economic asset exemplifies circular-economy principles in practice, positioning the citrus industry as a sustainability leader within the global food system.

The successful realisation of this roadmap requires coordinated action across multiple stakeholder groups: academic researchers providing fundamental insights, industry partners contributing operational expertise, government agencies establishing supportive policy frameworks, and investors providing capital for demonstration facilities [69,70]. International collaboration through research consortia and knowledge-sharing platforms accelerates progress whilst facilitating the dissemination of best practices essential to the implementation of commercial-scale lemon biorefineries.

## 8. Conclusions

This comprehensive review establishes that lemon-processing residues represent compositionally heterogeneous yet systematically exploitable matrices harbouring substantial concentrations of high-value compounds: essential oils (2.0–4.5% dry weight) rich in D-limonene, pectin (18–28%) with high methoxyl content suitable for food-gelling applications, bioactive polyphenols (84–139 mg GAE·g^−1^) exhibiting potent antioxidant and nutraceutical properties, seed oils (27–45%) with favourable fatty acid profiles, and cellulosic materials amenable to transformation into advanced nanomaterials commanding premium pricing (USD 50–150 per kg for nanocrystalline cellulose). The systematic bibliometric analysis of 847 publications spanning 2003–2025 documents exponential research growth, particularly post 2015, reflecting intensified policy emphasis on circular-economy principles and bio-based product development aligned with European Union and United Nations sustainability frameworks.

However, critical imbalances persist within the research landscape. Whilst extraction-methodology development dominates the literature—reflected in co-occurrence network analysis revealing prominent nodes for ultrasound-assisted extraction, microwave-assisted extraction, and supercritical fluid extraction—downstream processing, techno-economic-viability assessment, and industrial-scale implementation remain substantially underrepresented, with extraction-to-integration ratios exceeding 10:1. This disproportionate focus has generated extensive laboratory-scale optimisation data but insufficient guidance for commercial deployment, regulatory compliance, or market development—deficiencies that constrain translation from technical feasibility to industrial reality.

The comparative evaluation of green extraction technologies demonstrates substantial performance advantages over conventional approaches. Yield improvements of 16–112% are documented, depending on the target compound and methodology, whilst processing times decrease by 89–98% (from hours to minutes for microwave-assisted extraction) and energy consumption decreases by up to 95% (solventless microwave hydrodistillation versus conventional steam distillation). These technologies operate synergistically within cascade-biorefinery frameworks: supercritical CO_2_ or solventless microwave extraction for initial essential-oil recovery (1.5–2.5% yield, >95% limonene purity), ultrasound-assisted or enzyme-assisted extraction for polyphenol fractions (enhancing yields 30–50% over conventional methods), microwave-assisted or acid-based extraction for pectin isolation (10–30% yield with preserved functional properties), and subsequent cellulose processing yielding nanocrystalline cellulose from residual biomass. This sequential approach transforms disposal costs of USD 50–100 per tonne into potential revenues exceeding USD 500–1500 per tonne, depending on product-portfolio composition and market positioning, whilst reducing environmental impact by 60–80% relative to landfilling across greenhouse gas emissions, water pollution, and resource-depletion metrics.

Ten critical research gaps have been systematically identified through bibliometric network analysis and literature synthesis: (1) fragmentation of valorisation research prioritising single-product pathways over integrated cascade systems; (2) dominance of extraction studies at the expense of process integration and scale-up engineering; (3) limited attention to high-value emerging products, particularly nanocrystalline cellulose and α-cellulose derivatives; (4) insufficient integration of life-cycle assessment and techno-economic analysis, with fewer than 5% of publications incorporating comprehensive sustainability evaluation; (5) minimal industrial-implementation documentation and pilot-scale validation studies; (6) inadequate geographical and feedstock specificity, with compositional variability across cultivars and maturity stages insufficiently characterised; (7) limited market development and end-use application research; (8) underexplored biotechnological valorisation despite demonstrated advantages of biological processing; (9) regulatory-pathway clarification requirements for novel ingredients and nanomaterials; and (10) incomplete life-cycle assessment across diverse biorefinery configurations employing different technology combinations and allocation methodologies.

Techno-economic modelling across three implementation scenarios—small-scale distributed processing (50–100 tonnes per annum), medium-scale semi-continuous operations (500–1000 tonnes per annum), and large-scale integrated industrial biorefineries (5000–10,000 tonnes per annum)—demonstrates economic viability contingent upon cascade-valorisation principles and diversified product portfolios. Capital investment requirements range from USD 250,000–450,000 for small-scale facilities targeting pharmaceutical-grade niche products with 3–4 year payback periods, through USD 1.5–2.5 million for medium-scale operations achieving balanced product portfolios with 2–3 year payback to USD 8–15 million for large-scale biorefineries realising economies of scale through complete cascade valorisation including on-site enzyme production and anaerobic digestion with 2–3 year payback periods. Life-cycle assessment studies validate the environmental superiority of integrated biorefinery operations, documenting 60–80% reductions in global warming potential, eutrophication, and ecotoxicity impacts compared to conventional disposal, whilst preventing water pollution from D-limonene-containing leachates exhibiting phytotoxicity and aquatic toxicity.

The structured research roadmap delineated in Section 7.7 provides actionable guidance across three temporal horizons. Short-term priorities (1–2 years) emphasise pilot-scale technical validation of cascade protocols, development of pharmaceutical-grade quality specifications, a proactive regulatory engagement, publication of a peer-reviewed life-cycle assessment, and establishment of the first commercial demonstration facilities to document operational realities. Medium-term objectives (3–5 years) focus on scaling to continuous commercial operations with Industry 4.0 process control, establishing stable market channels through customer qualification and supply agreements, conducting comprehensive multi-site feedstock characterisation enabling cultivar-specific processing protocols, integrating renewable energy toward near-zero net-energy operations, and developing validated techno-economic models enabling investor decision-making. Long-term priorities (5–10 years) encompass establishment of 3–5 flagship biorefinery facilities achieving economies of scale and price competitiveness with synthetic alternatives, development of secondary applications for advanced materials and speciality chemicals, integration into circular-economy networks with adjacent food-processing industries, regulatory approval across major jurisdictions with harmonisation facilitating international commerce, and human clinical validation of health claims establishing evidence-based premium positioning.

The successful realisation of this vision requires coordinated action across multiple stakeholder groups and recognition that technological innovation alone proves insufficient without concurrent advances in market development, regulatory navigation, and infrastructure investment. Academic researchers must prioritise application-oriented investigations that address scale-up challenges and industrial-implementation barriers, rather than incremental laboratory-scale optimisation. Industry partners must transition from risk-averse disposal practices to value-creation mindsets, investing in demonstration facilities that generate operational knowledge whilst building investor confidence. Government agencies should establish supportive policy frameworks—research funding prioritising industrial translation, tax incentives rewarding the implementation of the circular economy, and procurement preferences favouring bio-based products—to accelerate commercial deployment. International collaboration through research consortia, pre-competitive technology-development programmes, and knowledge-sharing platforms accelerates progress whilst avoiding duplicative efforts and facilitating the dissemination of best practices.

The convergence of green extraction technologies with Industry 4.0 concepts—real-time process monitoring through in-line analytical sensors, machine learning algorithms that optimise multiparameter extraction protocols more efficiently than traditional response surface methodology, and digital twin modelling that enables virtual process development and operator training—promises revolutionary advances in industrial-scale citrus-waste valorisation. The emerging regulatory landscape, increasingly favouring naturally derived bioactive compounds over synthetic alternatives, driven by consumer clean-label preferences and mounting evidence of unique biological activities, creates favourable market conditions for biorefinery products. Policy frameworks, including the European Union’s Farm to Fork strategy, circular-economy action plans, and the United Nations Sustainable Development Goals, explicitly prioritise food-waste valorisation and the development of a bio-based economy, providing research funding, preferential market access, and public recognition that collectively incentivise sustainable practices.

The citrus-processing industry thus confronts a strategic inflexion point at which environmental-compliance mandates, consumer-demand trajectories, and economic opportunities converge, necessitating a transition from disposal-focused to valorisation-focused operational paradigms. The transformation of lemon-processing waste from liability—incurring disposal costs, environmental burdens, and foregone opportunities—to asset—generating diversified revenue streams, enhancing corporate sustainability profiles, and contributing to regional economic development—exemplifies circular-economy principles in practice. This review establishes that the technical foundations, financial incentives, and policy frameworks enabling this transformation are increasingly aligned, positioning the lemon-biorefinery concept for accelerated commercial deployment over the coming decade. Part II of this review series systematically addresses implementation practicalities by examining circular-economy integration strategies, industrial case studies documenting operational experiences, detailed techno-economic assessment methodologies, regulatory compliance pathways across major jurisdictions, and future perspectives on emerging technologies and market opportunities essential to realising the full potential of lemon-waste valorisation within sustainable, circular food-production systems.

## Figures and Tables

**Table 1:** Critical research gaps in lemon biorefinery development and associated research priorities.

Research Gap	Observation	Implication	Research Needs
1. Fragmentation of Valorisation Research and Lack of Integration	Research remains predominantly focused on single-product pathways. Individual products (essential oils, pectin, bioactive extracts) are extensively investigated in isolation, with sparse integration within unified cascade frameworks.	Fragmentation hinders the development of economically viable biorefineries requiring diversified revenue streams. Techno-economic analyses demonstrate the superior performance of cascade approaches [61,75].	Systematic investigations of cascade-valorisation sequences, quantifying synergies and trade-offs. Process simulation and techno-economic models evaluating complete cascade configurations.
2. Dominance of Extraction Studies over Process Integration	Research on extraction methodologies constitutes the most intensively studied area, at the expense of downstream processing, product purification, and scale-up engineering. The extraction-to-process-integration ratio exceeds 10:1.	Knowledge base is disproportionately weighted toward laboratory-scale optimisation, with insufficient attention to industrial viability challenges, including continuous processing, solvent recovery, and energy integration [76].	Engineering-focused research addressing scale-up challenges: continuous reactor design, intensified separation, energy-efficient drying. Pilot-scale validation studies. Alternative drying methods for nanocrystalline cellulose [67].
3. Limited Attention to High-Value, Emerging Products	Advanced products (nanocrystalline cellulose, α-cellulose, citric acid) are underrepresented despite commercial potential. Bias towards well-established, low-value applications. NCC shows minimal connectivity to the biorefinery cluster.	Most economically transformative products—NCC commanding prices exceeding GBP 100/kg—remain underdeveloped. Citrus-derived NCC demonstrates properties comparable to wood-derived materials (crystallinity 65–71%) [34,77].	Focused programmes on NCC production: process optimisation (yield, crystallinity), cost-reduction strategies (spray/supercritical drying), application development (nanocomposites, packaging, biomedical). α-cellulose production and derivatives.
4. Insufficient Integration of Techno- Economic and Environmental Assessment	LCA, TEA, economic viability, and sustainability metrics are notably absent or weakly represented. Less than 5% of publications incorporate a comprehensive economic or environmental evaluation.	Research remains technically focused, with limited consideration of economic feasibility or environmental impact, hampering industrial translation. Recent LCA studies identify hydrolysis and energy-intensive operations as major hotspots [74,77].	Integrated LCA-TEA studies of complete cascade systems: capital/operating costs, revenue projections, sensitivity analyses, comparative environmental performance. Regional assessments accounting for local conditions. “Cradle-to-grave” approaches.
5. Limited Industrial Implementation and Scale-Up	Minimal terminology related to industrial implementation (pilot plant, commercial scale, process control, regulatory compliance). Few case studies, predominantly at laboratory or small scale (≤10 tonnes/day).	Substantial gap between academic research (TRL 1–4) and commercial deployment (TRL 8-9). “Valley of death” refers to the challenges faced by novel products and integrated processes. Industrial pectin production was established, but integration was limited [73].	Documentation of pilot-scale and commercial facilities: operational challenges, performance data, economic outcomes. Industry–academic partnerships. Regulatory-pathway research (food safety, novel food status, nanomaterial regulations).
6. Insufficient Geographical and Feedstock Specificity	Research has disproportionately focused on Mediterranean varieties, with limited attention to regional variability. Compositional studies reveal pectin (15–25%) and essential oil (1–3%) range depending on variety, maturity, and conditions.	Optimised strategies may not transfer due to compositional variability, seasonal patterns, and differences in infrastructure. Assam lemon shows peak pectin content (3.07%) at 60 days, declining to 1.56% at 130 days [30,78].	Comparative studies across varieties (Eureka, Lisbon, Femminello, Primofiori, Assam) and regions, documenting compositional ranges and implications for yields. Adaptation strategies for different scales (rural facilities versus industrial complexes).
7. Lack of Market-Development and Application Research	Product extraction and characterisation are well studied, but end-use applications and market development receive minimal attention. Antioxidant activity is widely measured in vitro, but its commercial incorporation remains scarce.	Products remain “solution-seeking problems” rather than market responses. The disconnect between supply-push research and demand-pull innovation hinders commercialisation. The global pectin market (USD 1.4–1.6 billion) shows successful integration [67].	Application-focused research: specific formulations, end-use validation, consumer acceptance, shelf-life assessments. Partnerships between biorefinery researchers and product formulators are essential.
8. Limited Exploration of Biotechnological Valorisation	Biotechnological approaches remain secondary to physico-chemical extraction. Enzyme-assisted extraction, microbial production, and biocatalytic transformations are underexplored relative to their potential.	Biological processes offer advantages (mild conditions, selectivity, green chemistry) but remain underdeveloped. EAE demonstrates 65–88% pectin recovery with lower effluent volumes, but enzyme costs and longer times remain barriers [79,80].	Enzyme engineering for improved hydrolysis; microbial cell factories for limonene/citric acid conversion to biochemicals; anaerobic digestion optimisation (addressing D-limonene inhibition). Co-digestion strategies combining extraction with biogas production [81].
9. Regulatory-Pathway Clarification for Novel Ingredients and Nanomaterials	Nanocrystalline cellulose, novel enzyme formulations, and molecularly standardised bioactive extracts currently navigate uncertain regulatory terrain across jurisdictions (EU, USA, China). Regulatory clarity remains a commercialisation barrier.	Companies investing in nanocrystalline cellulose or enzyme-derived products face regulatory prosecution risk, which may require expensive reformulation or geographic market restriction.	Engage regulatory agencies (EFSA, FDA, CFDA) to establish science-based approval pathways; conduct safety and efficacy studies that meet regulatory standards; and develop dossiers for regulatory submission.
10. Limited Life-Cycle Assessment and Circular-Economy Quantification	Whilst fragmented LCA studies exist, comprehensive cradle-to-grave assessments comparing alternative valorisation scenarios and baseline landfilling across diverse biorefinery configurations remain scarce.	Companies pursuing sustainability certification (ISO 14040 [82], PAS 2050, Cradle to Grave approach [83,84]) lack sufficient data to substantiate environmental claims. The absence of a standardised LCA methodology impedes comparative marketing and investor confidence.	Conduct ISO 14040-compliant LCA studies for 3–5 representative biorefinery scenarios; develop transparent LCA databases; establish industry-consensus allocation methodologies for multi-product systems.

**Table 2:** Chemical composition of lemon-processing residue fractions.

Component	Flavedo	Albedo	Seeds	Pomace	Unit	References
Proximate Composition						
Moisture ^a^	70–76	65–70	45–55	75–85	%	[85]
Ash ^b^	3.0–4.5	3.5–5.0	4.0–6.0	3.0–5.0	%	[85]
Protein ^b^	4–7	5–9	8–15 ^c^	4–8	%	[86]
Lipids and Essential Oils						
Essential-Oil Content	2.0–4.5	0.2–0.5	0.5–1.2	0.3–0.8	% DW ^d^	[87,88]
Extractable Oil	-	-	27–45	-	% DW	[89]
Limonene	60–76	35–50	8–15	40–60	% EO ^e^	[87,90]
β-Pinene	8–12	5–8	3–6	6–10	% EO	[88]
γ-Terpinene	6–10	4–7	2–5	5–8	% EO	[87]
Structural Carbohydrates						
Cellulose	8–12	15–22	10–16	12–18	% DW	[86]
Hemicellulose	4–7	8–14	6–10	6–10	% DW	[86]
Lignin	1–3	0.5–2.0	8–12	3–6	% DW	[86]
Pectin	12–18	18–28	2–5	8–15	% DW	[30,35]
Soluble Sugars						
Glucose	2–5	1–4	1–3	3–7	% DW	[51]
Fructose	2–5	1–4	1–3	3–7	% DW	[51]
Sucrose	1–3	0.5–2.0	0.5–2.0	1–4	% DW	[51]
Organic Acids						
Citric Acid	0.5–1.5	0.3–1.0	0.2–0.8	8–15	% DW	[91,92]
Phenolic Compounds						
Total Phenolics	102–139	84–120	15–35	25–45	mg GAE/g DW	[85]
Hesperidin	25–45	35–65	1.2–2.5	8–15	mg/g DW	[85,93]
Eriocitrin	8–18	12–28	0.3–1.0	3–8	mg/g DW	[87]
Naringin	3–8	5–12	0.5–1.5	2–6	mg/g DW	[85]
Tangeretin (PMF) ^f^	2–6	0.1–0.5	ND ^g^	0.2–0.8	mg/g DW	[93]
Sinensetin (PMF)	1–4	0.1–0.3	ND	0.1–0.5	mg/g DW	[93]
Seed-Specific Components						
Oleic Acid (C18:1)	-	-	24–32	-	% tFA ^h^	[89]
Linoleic Acid (C18:2)	-	-	34–42	-	% tFA	[89]
Palmitic Acid (C16:0)	-	-	18–24	-	% tFA	[89]
Functional Properties						
Water-holding capacity	4.2–6.8	8.5–10.9	-	5.5–8.2	g/g	[85]
Oil-binding capacity	2.8–4.5	5.2–6.3	-	3.5–5.0	g/g	[85]

^a^ Fresh basis. ^b^ Dry basis. ^c^ Protein content in defatted seed meal. ^d^ Dry-weight basis. ^e^ Essential Oil. ^f^ Polymethoxylated flavone. ^g^ Not Determined. ^h^ Total Fatty Acid.

**Table 3:** Variability of key bioactive compounds across major lemon cultivars (dry-weight basis).

Cultivar	Origin	Essential Oil (Limonene %)	Pectin Yield (%)	Hesperidin (mg·g^−1^)	References
Eureka	Global	65–70	22.8–28.0	18.5–24.0	[113,114]
Lisbon	USA/Portugal	60–68	20.0–25.0	15.0–22.0	[113,115]
Femminello	Italy	68–75	24.0–29.0	22.0–35.0	[116,117]
Verna	Spain	55–65	18.0–22.0	12.0–18.0	[113,118]

**Table 4:** Typical products and process streams in lemon cascade valorisation.

Fraction	Major Bioproducts	Typical Extraction Method	Industrial Application	References
Essential oils	Limonene, alpha-terpineol	Cold press, distillation	Flavours, cosmetics, therapeutics	[130,131,133,152]
Peel, rag	Pectin, polyphenols	Acid/ enzyme extraction	Food, pharmaceuticals, dietary supplements	[30,31,134,135,153]
Seeds, pomace	Proteins, dietary fibres	Solvent/ enzymatic	Animal feed, functional foods	[34,89,154,155,156]
Aqueous effluent	Polyphenols, organic acids	Membrane/ adsorption	Nutraceuticals, food preservatives	[157,158]
Residue	Bioethanol, biogas, fertilisers	Fermentation, composting	Renewable energy, soil amendments	[61,126,159]

**Table 5:** Primary valorisation pathways for lemon-processing residues: extraction technologies, yields, industrial applications, and key references.

Pathway	Compounds	Source Fraction	Extraction Technology	Optimal Conditions	Typical Yield	Purity/Quality	Industrial Applications	Market Value (USD/kg)	Ref.
Essential Oils and D-limonene	D-limonene, β-pinene, γ-terpinene	Flavedo	Cold pressing, hydrodistillation	100 °C, 2–4 h	0.5–5.0% FW ^a^	Limonene: 70–95%	Flavours, fragrances, cosmetics	15–30	[160,161]
	D-limonene, monoterpenes	Flavedo	Microwave- assisted (MAE)	360 W, 1–3 min, solvent-free	2.0–3.5% DW ^b^	Limonene: 65–79%	Flavours, fragrances	15–30	[130,162]
	D-limonene, terpenes	Flavedo	Ultrasound- assisted (UAE)	20–25 kHz, 60 °C, 20 min	32.9 mg/g DW	Limonene: 95–97%	Premium applications	15–30	[54]
	D-limonene, sesquiterpenes	Flavedo	Supercritical CO_2_	100–200 bar, 40–60 °C, 2–5 h	1.5–2.5%	Limonene: 95–99%	Pharmaceutical-grade	30–80	[163,164]
	D-limonene, terpenes	Flavedo	Bio-based solvent extraction	CPME ^e^, 2-MeTHF ^f^, ambient	40–80% higher than hexane	Limonene: 85–95%	Food-grade, sustainable	20–40	[165]
Pectin	High methoxyl pectin, galacturonic acid	Albedo, peel	Conventional acid extraction	pH 1.5–3.0, HCl, 60–100 °C, 30–120 min	18–35% DW	DE ^c^: 55–75%, GalA ^d^: 61–74%	Gelling agents, stabilisers	8–15	[166,167]
	High methoxyl pectin	Albedo, peel	Microwave- assisted (MAE)	360 W, pH 2.2, 1–10 min, pulse	7.6–18%	DE: 66.7%, GalA: 63.2%	Food gelling, pharmaceuticals	8–15	[168,169]
	High methoxyl pectin	Albedo, peel	Ultrasound- assisted (UAE)	60–75 °C, 15–45 min, citric acid	10–17%	DE: 55.3%	Biodegradable packaging	8–15	[37]
	High methoxyl pectin	Albedo, peel	Pulsed electric field (PEF)	80 °C, 9 V/cm, 30–60 min	14–18%	Excellent emulsifying	Active packaging	10–18	[170]
	High methoxyl pectin	Albedo, peel	Citric acid extraction	pH 2.2, 80 °C, 50 min	32.5%	DE: 66.4%, methoxyl: 7.7%	Food-grade, clean-label	10–16	[171,172]
	Pectin with polyphenols	Albedo, peel	Deep eutectic solvent (DES)	Citric acid–glycerol DES ^h^, UAE	Variable	Comprehensive valorisation	Integrated biorefinery	10–18	[35]
Seed Oil	Oleic (21–44%), linoleic (31–48%)	Seeds	Solvent extraction (Soxhlet)	Hexane, 60 °C, 6–8 h	71.3% of seed oil	α-tocopherol: 110 mg/kg	Edible oils, biodiesel	5–15	[156]
	Unsaturated fatty acids, tocopherols	Seeds	Cold pressing	Ambient, mechanical	36.8% of seed oil	α-tocopherol: 155 mg/kg	Premium oils, cosmetics	15–30	[89,154]
	Fatty acids, phenolics, flavonoids	Seeds	Supercritical CO_2_	200–350 bar, 40–55 °C	Variable	Total phenolics: 165.9 mg/mL	Pharmaceutical, nutraceuticals	30–80	[89]
	Fatty acid methyl esters (FAME)	Seeds	Transesterification	Alkaline catalyst, 60 °C	94% conversion	Meets ASTM D6751, EN 14214	Biodiesel	5–15	[173]
Citric Acid	Citric acid	Peel, pomace	SSF ^i^ (*A. niger*)	28 °C, pH 4.5–6.5, 5–7 days	193.2 mg/g DW	Fermentation-grade	Food acidulant, pharmaceuticals	1–3	[174]
	Citric acid	Peel, pomace	SubF ^j^ (*A. niger*)	Hydrolysates, pH 4.5–6.5, 28–30 °C	Variable	Industrial-grade	Food processing	1–3	[175]
	Citric acid	Peel, pomace	Fermentation (*Y. lipolytica*)	28 °C, pH 5–6, glucose/acetate	72.3 g/L (glucose); 15.1 g/L (acetate)	Yield: 0.77 g/g; 0.51 g/g	Versatile substrates	1–3	[176]

^a^ FW: fresh weight; ^b^ DW: dry weight; ^c^ DE: degree of esterification; ^d^ GalA: galacturonic acid; ^e^ CPME: cyclopentyl methyl ether; ^f^ 2-MeTHF: 2-methyltetrahydrofuran; ^h^ DES: deep eutectic solvent; ^i^ SSF: solid-state fermentation; ^j^ SubF: submerged fermentation.

**Table 6:** Comparative technologies for the synthesis of cellulosic materials from lemon waste.

Product	Method	Key Conditions	Characteristics and Yield	Ref.
α-Cellulose	Alkaline/Oxidative Treatment	NaOH (4–8%), H_2_O_2_/ NaClO, 60–100 °C	High purity (>85%), crystallinity 65–70%. Low lignin content simplifies purification.	[193]
	Hydrodynamic Cavitation	Water-based, controlled bubble collapse	“CytroCell”: Low crystallinity (45–55%), high porosity. Chemical-free, “green” process.	[74,194]
NCC *	Sulfuric Acid Hydrolysis	60–65 wt% H_2_SO_4_, 45–60 °C, 20–60 min	Negatively charged surface (sulfate esters). High yield (17–19%) but lower thermal stability.	[195,196]
	Ammonium Persulfate Oxidation	(NH_4_)_2_S_2_O_8_, 60–80 °C	Carboxylated surface (high stability). Highest crystallinity (~73.8%) of lemon NCC.	[195,197]
	TEMPO-Mediated Oxidation	pH 10–11, room temperature	High yield (~19%). Mild conditions preserve C6 positions for functionalisation.	[197,198]
	Enzymatic Hydrolysis	Cellulases, pH 4.5–5.5, 40–50 °C	Eco-friendly, no surface charge introduction. Longer processing time (24–72 h).	[195]

* NCC: nanocrystalline cellulose (also termed cellulose nanocrystals, CNCs).

**Table 7:** Advanced applications of lemon-derived nanocellulose based on functional properties.

Application Field	Function of NCC	Mechanism/Benefit	Ref.
Nanocomposites	Reinforcement Agent	High aspect ratio improves tensile strength and elastic modulus in PLA/PVA matrices.	[200,201]
Active Packaging	Barrier Film	High crystal clearness creates tortuous paths, thereby reducing the permeability for oxygen and water vapour.	[202,203]
Food and Cosmetics	Pickering Emulsifier	An amphiphilic surface stabilises oil/water interfaces without synthetic surfactants.	[204]
Biomedical	Scaffold/Carrier	Biocompatibility and tailorable surface chemistry allows drug delivery and tissue engineering.	[205,206]
Rheology	Modifier/Thickener	Rod-like morphology forms thixotropic gels via hydrogen bonding networks.	[207]

**Table 8:** Biotechnological valorisation pathways for lemon-processing residues: microbial bioconversion, bioenergy production, and biological stabilisation.

Valorisation Pathway	Process/ Organism	Substrate Fraction	Optimal Conditions	Product Yield	Quality Parameters	Applications	Ref.
Microbial Biomass for Food/Feed							
Single-cell protein	*Saccharomyces cerevisiae*, *Candida utilis*	Hydrolysed peel sugars	28–30 °C, pH 4.5–5.5, aerobic	0.4–0.5 g·g^−1^ sugar	45–55% crude protein, essential amino acids	Animal feed, aquaculture	[141,145]
Probiotic biomass	*Lactobacillus* spp.	Citrus pomace	37 °C, pH 6.0–6.5, anaerobic	10^9^–10^10^ CFU·mL^−1^	Viable counts, acid tolerance	Functional foods, feed additives	[145]
Fungal protein	*Aspergillus oryzae*, *Rhizopus oligosporus*	De-oiled peel (SSF) ^1^	28–32 °C, 72–120 h, 60–70% moisture	18–25% protein enrichment	Improved digestibility, reduced anti-nutrients	Ruminant feed, fermented foods	[141,143]
Bioenergy Production							
Bioethanol	*S. cerevisiae*, *Kluyveromyces marxianus*	Detoxified hydrolysates	30–37 °C, pH 5.0, 48–72 h	35–45 L· tonne^−1^ FW ^2^	85–92% theoretical yield, >99% purity (distilled)	Transport fuel, industrial solvent	[54,72]
Biomethane	Anaerobic consortia	Delimonened residues	35–55 °C, pH 6.8–7.5, HRT ^3^ 20–30 d	450–550 mL CH_4_·g^−1^ VS ^4^	60–70% CH_4_, <200 ppm H_2_S	CHP ^9^ systems, grid injection	[72,146]
Biohydrogen	*Clostridium* spp., mixed cultures	Citrus-processing effluents	35–37 °C, pH 5.5–6.5, dark fermentation	85–120 mL H_2_·g^−1^ COD ^5^	>90% H_2_ purity	Fuel cells, chemical synthesis	[150,159]
Platform Chemicals							
Volatile fatty acids (VFA)	Mixed acidogenic cultures	Peel hydrolysates	35 °C, pH 5.5–6.5, 5–10 d	0.3–0.5 g VFA·g^−1^ VS	Acetate/propionate/butyrate (6:2:2)	PHA ^6^ precursors, chemical synthesis	[126]
Succinic acid	*Actinobacillus* *succinogenes*	Glucose from hydrolysis	37 °C, pH 6.8, CO_2_-enriched	25–35 g·L^−1^	>90% purity, 0.7–0.8 g·g^−1^ yield	Bioplastics, PBS ^7^ synthesis	[61]
D-Limonene biotransformation	*Penicillium* *digitatum*	Essential-oil fraction	25–28 °C, 5–7 d	α-Terpineol: 3–5 g·L^−1^	>95% regioselectivity	Fragrances, pharmaceuticals	[141]
Biological Stabilisation							
Aerobic composting	Indigenous microbiota + inoculants	Mixed citrus residues	55–65 °C thermophilic, 8–12 wks.	40–50% mass reduction	C:N 15–20, mature compost	Soil amendment, horticulture	[149]
Vermicomposting	*Eisenia fetida* + microbiota	Pre-composted peel	20–25 °C, 60–70% moisture, 8–10 wks.	0.4–0.5 kg·kg^−1^ input	NPK-enriched, humic acids	Organic fertiliser, potting media	[151]
Anaerobic digestate	Post-AD ^8^ residual solids	Digester effluent	Dewatering, stabilisation	15–25 kg· tonne^−1^ input (dry)	Mineralised N, P, K; low pathogens	Biofertiliser, soil conditioner	[61,146]

^1^ SSF: solid-state fermentation; ^2^ FW: fresh weight; ^3^ HRT: hydraulic retention time; ^4^ VS: volatile solids; ^5^ COD: chemical oxygen demand; ^6^ PHA: polyhydroxyalkanoates; ^7^ PBS: polybutylene succinate; ^8^ AD: anaerobic digestion; ^9^ CHP: combined heat and power.

**Table 9:** Comparative performance metrics between conventional and green technologies.

Dimension	Conventional	UAE ^1^	MAE ^2^	SC-CO_2_ ^3^	EAE ^4^
Yield increase (%)	60–75	85–95	75–90	90–98	70–85
Energy consumption (kWh·kg^−1^)	10–15	0.8–1.2	0.3–0.5	2.5–4.0	0.5–0.9
Processing time	8–16 h	20–60 min	1–10 min	2–5 h	8–24 h
Solvent consumption (L·kg^−1^)	3–6	2–5	1–3	0	1.5–1.0
Solvent toxicity	High– Moderate	Low– Moderate	Minimal	None	Minimal
Product quality (thermal degradation risk)	High (80–100 °C)	Moderate (40–75 °C)	Low (40–80 °C)	Minimal (<50 °C)	Minimal (40–50 °C)
Bioactive preservation ^5^	60–75%	85–95%	85–98%	95–99%	95–98%
Scalability level	Excellent	Good	Excellent	Moderate	Moderate– Good
Waste Generation	Moderate– High	Low– Moderate	Low	Minimal	Low
GWP ^6^ reduction vs. conventional	Baseline	65–78%	80–92%	71–89%	70–85%
Capital cost ^7^ (× 10^3^ USD)	10–50	50–600	60–700	300–4000	40–150
Operating cost ^7^ (USD per tonne)	120–180	130–200	91–139	165–250	110–180
References	[244,245]	[237,246]	[247,248]	[38,249]	[80,250]

^1^ UAE: ultrasound-assisted extraction; ^2^ MAE: microwave-assisted extraction; ^3^ SC-CO_2_: supercritical CO_2_ extraction; ^4^ EAE: enzymatic-assisted extraction; ^5^ *Bioactive preservation* refers to the retention percentage of thermolabile compounds (e.g., polyphenols, flavonoids, vitamins) after extraction; ^6^ GWP: global warming potential (in terms of kg CO_2_ equivalent per functional unit); ^7^ Capital and operating costs are approximate estimates (in USD, 2020–2024) derived from techno-economic assessments [38,246,248]; values may vary with production scale, location, and market conditions.

## Data Availability

No new data were created or analyzed in this study. Data sharing is not applicable to this article.

## Abbreviations

The following abbreviations are used in this manuscript:

ABTS	2,2′-Azino-bis(3-ethylbenzothiazoline-6-sulphonic acid)
ASTM	American Society for Testing and Materials
BHA	Butylated Hydroxyanisole
BHT	Butylated Hydroxytoluene
CMC	Carboxymethyl Cellulose
CNC	Cellulose Nanocrystals
CPME	Cyclopentyl Methyl Ether
CUPRAC	Cupric-Ion-Reducing Antioxidant Capacity
DES	Deep Eutectic Solvents
DLS	Dynamic Light Scattering
DPPH	2,2-Diphenyl-1-picrylhydrazyl
DW	Dry Weight
EAE	Enzyme-Assisted Extraction
EDTA	Ethylenediaminetetraacetic Acid
EN	European Norm
FESEM	Field-Emission Scanning Electron Microscopy
FRAP	Ferric-Reducing Antioxidant Power
FTIR	Fourier Transform Infrared Spectroscopy
GAE	Gallic Acid Equivalents
GRAS	Generally Recognised as Safe
HM	High Methoxyl
LCA	Life-Cycle Assessment
LM	Low Methoxyl
MAE	Microwave-Assisted Extraction
MCC	Microcrystalline Cellulose
MDA	Malonaldehyde
MeTHF	Methyltetrahydrofuran
MIC	Minimum Inhibitory Concentration
NaDES	Natural Deep Eutectic Solvents
NCC	Nanocrystalline Cellulose
NFC	Nanofibrillated Cellulose
PEF	Pulsed Electric Field
PLA	Polylactic Acid
PMF	Polymethoxylated Flavones
PVA	Polyvinyl Alcohol
RSM	Response Surface Methodology
SC-CO2	Supercritical Carbon Dioxide
SCP	Single-Cell Protein
SSF	Solid-State Fermentation
TEA	Techno-Economic Analysis
TEMPO	2,2,6,6-Tetramethylpiperidine-1-oxyl
TGA	Thermogravimetric Analysis
TPC	Total Phenolic Content
UAE	Ultrasound-Assisted Extraction
UAEE	Ultrasound-Assisted Enzymatic Extraction
XPS	X-ray Photoelectron Spectroscopy
XRD	X-ray Diffraction
